# Seven new species of Night Frogs (Anura, Nyctibatrachidae) from the Western Ghats Biodiversity Hotspot of India, with remarkably high diversity of diminutive forms

**DOI:** 10.7717/peerj.3007

**Published:** 2017-02-21

**Authors:** Sonali Garg, Robin Suyesh, Sandeep Sukesan, SD Biju

**Affiliations:** 1 Systematics Lab, Department of Environmental Studies, University of Delhi, Delhi, India; 2 Kerala Forest Department, Periyar Tiger Reserve, Kerala, India

**Keywords:** Amphibians, Ancient lineage, Bioacoustics, DNA barcoding, Endemism, Integrative systematics, Miniature frogs, *Nyctibatrachus*, Species diversity, Western Ghats

## Abstract

The Night Frog genus *Nyctibatrachus* (Family Nyctibatrachidae) represents an endemic anuran lineage of the Western Ghats Biodiversity Hotspot, India. Until now, it included 28 recognised species, of which more than half were described recently over the last five years. Our amphibian explorations have further revealed the presence of undescribed species of Nights Frogs in the southern Western Ghats. Based on integrated molecular, morphological and bioacoustic evidence, seven new species are formally described here as *Nyctibatrachus athirappillyensis* sp. nov., *Nyctibatrachus manalari* sp. nov., *Nyctibatrachus pulivijayani* sp. nov., *Nyctibatrachus radcliffei* sp. nov., *Nyctibatrachus robinmoorei* sp. nov., *Nyctibatrachus sabarimalai* sp. nov. and *Nyctibatrachus webilla* sp. nov., thereby bringing the total number of valid *Nyctibatrachu*s species to 35 and increasing the former diversity estimates by a quarter. Detailed morphological descriptions, comparisons with other members of the genus, natural history notes, and genetic relationships inferred from phylogenetic analyses of a mitochondrial dataset are presented for all the new species. Additionally, characteristics of male advertisement calls are described for four new and three previously known species. Among the new species, six are currently known to be geographically restricted to low and mid elevation regions south of Palghat gap in the states of Kerala and Tamil Nadu, and one is probably endemic to high-elevation mountain streams slightly northward of the gap in Tamil Nadu. Interestingly, four new species are also among the smallest known Indian frogs. Hence, our discovery of several new species, particularly of easily overlooked miniaturized forms, reiterates that the known amphibian diversity of the Western Ghats of India still remains underestimated.

## Introduction

The mountains of the Western Ghats in Peninsular India are a global biodiversity hotspot (Myers et al., 2000; Mittermeier et al., 2004). Over 92% of the presently known 225 amphibian species (Frost, 2016) of the Western Ghats are endemic to this region. An exponential two-fold increase in the number of species over the last decade and a half (2001–2015) has evidenced that a vast majority of amphibians of this region had remained undiscovered until recently (Biju, 2001), and the trends also suggest that the actual diversity may still be higher than currently estimated (Biju et al., 2014a). The present rate of species description from the Western Ghats is also comparable to the eight ‘hottest’ biodiversity hotspots of the world (Myers et al., 2000). Between 2006–2015, 1,581 new species of amphibians were described globally (AmphibiaWeb, 2016). Of these maximum are from the Brazilian Atlantic Forest (approximately 182) and the second highest from the Western Ghats-Sri Lanka (approximately 159), as per estimates based on AmphibiaWeb (2016), Frost (2016) and Conservation International (2016). The Western Ghats alone also stands at a close fourth position (with 103 new species) after Indo-Burma (approximately 117). Therefore, the Western Ghats can be considered among the leading biodiversity hotspot regions in terms of the number of new amphibian species described over the past decade. Such rapid rates of species discoveries can be attributed to intensified explorations as well as the increased use of molecular tools and integrated systematic approaches (e.g., Glaw & Köhler, 1998; Hanken, 1999; Hebert et al., 2003; Köhler et al., 2005; Vieites et al., 2009). These have also aided comprehensive taxonomic revisions of various amphibian groups in the Western Ghats (e.g., Biju & Bossuyt, 2009; Biju et al., 2011; Gower et al., 2011; Biju et al., 2014a; Biju et al., 2014b) thereby providing stable and reliable data for further identification of new taxa.

The anuran family Nyctibatrachidae is considered to be an ancient lineage of frogs that originated on the Indian landmass between the Cretaceous (Middle to Upper) and Palaeocene periods, synchronous with the long period of its isolation from the Gondwana (Roelants, Jiang & Bossuyt, 2004). This relic family comprises of two genera—*Lankanectes* Dubois & Ohler, 2001 and *Nyctibatrachus* Boulenger, 1882—which are restricted to Sri Lanka and the Western Ghats of India, respectively. Since the former is a monotypic genus with a single known species (*Lankanectes corrugatus*) and the latter includes 28 species recognised as valid (Frost, 2016), family Nyctibatrachidae can largely be considered as a radiation of the Western Ghats. Members of the genus *Nyctibatrachus*, commonly known as Night Frogs (Frank & Ramus, 1995) are known to occur in five states in southern India, with their distribution extending from the southern tip of Indian Peninsula in Tamil Nadu to Kerala, Karnataka, Goa and ending in northern Maharashtra. These frogs can be found in close association with mountain streams or marshes in forests of the Western Ghats (Biju et al., 2011). Their sizes vary from 10–77 mm (Snout-vent length) (Biju et al., 2011), including the 10 mm long *Nyctibatrachus minimus*, which is the smallest Indian tetrapod described so far (Biju et al., 2007).

The Night Frog genus *Nyctibatrachus* is among the relatively well-studied amphibian groups of the Western Ghats. The first *Nyctibatrachus* species was described in 1882 along with the recognition of the genus (Boulenger, 1882). Over the next century, another nine species were described (Bhaduri & Kripalani, 1955; Dubois, 1984; Inger et al., 1984; Myers, 1942; Rao, 1920; Rao, 1937; Ravichandran, 1997), and this number was more than doubled by new descriptions during the past decade (Biju et al., 2007; Biju et al., 2011; Das & Kunte, 2005; Dinesh, Radhakrishnan & Bhatta, 2008; Gururaja et al., 2014). A comprehensive systematic revision by Biju et al. (2011) provided taxonomic stability for various previously available names in this genus. The latest studies on *Nyctibatrachus* frogs have particularly drawn attention towards their natural history, including reproductive behaviour and ecology (e.g., Kunte, 2004; Biju et al., 2011; Gramapurohit, Gosavi & Phuge, 2011; Gururaja et al., 2014; Willaert et al., 2016) and tadpole development (Annandale, 1918; Annandale, 1919; Rao, 1923; Bhaduri & Kripalani, 1955; Pillai, 1978; Priti, Gururaja & Ravikanth, 2015), as well as patterns of distribution and endemism in the Western Ghats (Van Bocxlaer et al., 2012). Male advertisement calls have so far been described for *N. major* (Kuramoto & Joshy, 2001), *N. jog*, *N. kempholeyensis*, *N. kumbara* (Gururaja et al., 2014) and *N. humayuni* (Willaert et al., 2016). In the case of *N. humayuni*, the rare occurrence of female calls has also been documented (Willaert et al., 2016).

During our routine amphibian surveys in the Western Ghats (in 2002 and between 2013–2016), we sampled various populations of *Nyctibatrachus* from the southern Western Ghats states of Kerala and Tamil Nadu, which reveal undescribed diversity of Night Frogs. Based on newly collected material, molecular, morphological and bioacoustic data, here we describe seven new species of *Nyctibatrachus* from the Western Ghats of India.

## Materials and Methods

### Field surveys and specimen collection

Field surveys and collection of specimens were carried out in 2002 and between 2013–2016 in forests of Kerala and Tamil Nadu, in southern Western Ghats, India. Adult specimens were collected during both day and night, mostly by locating calling males and sometimes through opportunistic surveys. Geographical coordinates of the sampling localities were recorded on a Garmin 76CSx GPS using the WGS-84 datum. Live animals were photographed, euthanised in MS-222 (Tricaine methane sulphonate), fixed in 4% formalin and preserved in 70% ethanol. Prior to fixation, a portion of the thigh muscle was preserved in absolute ethanol for subsequent molecular analyses. Tissue samples were stored at −20 °C in the Systematics Lab, University of Delhi. Type specimens are deposited in the Zoological Survey of India–Western Ghats Regional Centre (ZSI-WGRC), Calicut, and referred specimens are available at the Systematics Lab, University of Delhi (SDBDU), India. The study was conducted with permissions and following guidelines from the responsible authorities in the State Forest Departments of Kerala and Tamil Nadu (Study permits: No.WL12-1830/2009, No.WL10-2606/12, No.WL10-25421/2014, No.67254/2001/WL5). Research received ethical approval from Department of Environmental Studies, University of Delhi (DES/1020 dated 9 February 2015), India.

Abbreviations for museums and frequently used terms are as follows: SDBDU (Systematics Lab, University of Delhi, India), ZSI/WGRC (Zoological Survey of India, Western Ghats Regional Centre, formerly Zoological Survey of India, Western Ghats Field Research Station, WGFRS, Calicut, India), ZSIC (Zoological Survey of India, Kolkata, India), BNHS (Bombay Natural History Society, Bombay, India), NHM (Natural History Museum, formerly British Museum Natural History, BMNH, London, United Kingdom), IUCN (The International Union for Conservation of Nature), SDB (S.D. Biju) and SG (Sonali Garg).

### Molecular study

Genomic DNA was extracted from ethanol-preserved tissue samples using the Qiagen DNeasy blood and tissue kit (Qiagen, Valencia, CA, USA) following manufacturer’s protocol. A fragment of the mitochondrial 16S rRNA gene (540 bp) was PCR-amplified with primer sets 16Sar and 16Sbr published by Simon et al. (1994), and sequenced in both directions using the BigDye Terminator v3.1 Cycle Sequencing kit (Applied Biosystems) on ABI 3730 automated DNA sequencer (Applied Biosystems). Sequences were checked and assembled in ChromasPro v1.34 (Technelysium Pty Ltd.). A DNA matrix was assembled for 36 taxa, representing 28 previously known and seven new *Nyctibatrachus* species, and *Lankanectus corrugatus* as the outgroup taxon. Previously published 16S mitochondrial gene sequences for known species (Van Bocxlaer et al., 2012; Gururaja et al., 2014) were retrieved from the GenBank and new sequences generated as part of this study are deposited under accession numbers KY447300 –KY447307. The details of sequences used in the study are provided in Table S1.

Alignment was created in MEGA 6.0 (Tamura et al., 2013) using the ClustalW tool and manually optimised. The resultant DNA matrix (540 bp) was executed in PAUP* 4.0b10 (Swofford, 2002) to construct a Neighbour-Joining (NJ) tree by the Kimura 2-parameter (K2P) distance model, in order to verify species identification and to estimate species diversity. Uncorrected pairwise distances were also computed in PAUP* 4.0b10 for delineation of species. Phylogenetic relationships were estimated under the Maximum likelihood (ML) criteria and an appropriate model of DNA evolution was determined by implementing the Akaike Information Criterion in ModelTest 3.4 (Posada & Crandall, 1998). Heuristic ML searches were executed in PAUP* 4.0b10 using the best-fit model (GTR + G + I) and obtained parameters. Clade support was assessed with 1000 rapid bootstrap replicates executed under the likelihood framework using RAxML 7.3.0 (Stamatakis, 2006; Stamatakis, 2008), as implemented in raxmlGUI 1.1 (Silvestro & Michalak, 2012). Bayesian analyses were performed using the same model in MrBayes 3.1.2 (Ronquist & Huelsenbeck, 2003), with two parallel runs of four MCMC chains executed for 2 million generations. Trees were sampled after every 100 generations and the first 25% were discarded as burn-in. Bayesian Posterier Probabilities (BPP) for clades were estimated from a single 50% majority rule consensus tree.

### Morphological study

We used an integrated molecular and morphological approach to identify our collections. Based on genetic comparison with all the previously known 28 *Nyctibatrachus* species, candidate species were morphologically compared with the available type specimens and other referred museum specimens of closely related members within the genus. Sex and maturity were determined by the presence of secondary sexual characters such as nuptial pads, femoral glands and vocal sacs in males, or by examining the gonads through a small lateral or ventral incision. However, the presence or absence of femoral glands was not considered as a diagnostic character for species identification (Biju et al., 2011). Measurements and associated terminologies follow Biju et al. (2011). The term shank is used here to refer to the part of the leg that contains the tibia, and thigh is used for the part of the leg that contains the femur. The following measurements were taken to the nearest 0.1 mm using a digital slide caliper or a binocular microscope with a micrometer ocular: SVL (snout–vent length), HW (head width, at the angle of the jaws), HL (head length, from rear of mandible to tip of snout), SL (snout length, from tip of snout to anterior orbital border), EL (eye length, horizontal distance between bony orbital borders), EN (distance from the front of the eye to the nostril), NS (distance from the nostril to the tip of the snout), IUE (inter upper eyelid width, the shortest distance between the upper eyelids), UEW (maximum upper eyelid width), FAL (forearm length, from flexed elbow to base of outer palmar tubercle), HAL (hand length, from base of outer palmar tubercle to tip of third finger), FD (disc width of finger), FW (width of finger, measured at the base of the disc), SHL (shank length), TL (thigh length), FOL (foot length, from base of inner metatarsal tubercle to tip of fourth toe), TD (disc width), TW (width of toe, measured at the base of the disc). All measurements provided in the taxonomy section are in millimetres. Finger and toe disc morphological types follow Biju et al. (2011). All images of digit tips were taken with a Q Imaging camera through a Nikon SMZ 1500 microscope. The webbing formulae follow Savage & Heyer (1967) as modified by Myers & Duellman (1982). The amount of webbing relative to subarticular tubercles is described by numbering the tubercles 1–3, starting from the toe discs. All measurements and photographs were taken for the right side of the specimen, except when a character was damaged, in which case the measurement was taken on the left side. For the convenience of discussion, *Nyctibatrachus* species were grouped as miniature (male SVL 10–18 mm), small (male SVL 19–25 mm), medium (male SVL 26–40 mm), and large (male SVL 41–77 mm).

Discriminant function analysis (DFA) using Principal Component Analysis (PCA) factors were conducted to assess the degree of morphological differentiation among the new species and their closest relatives. Morphometric data for previously known members of the genus is taken from all the samples reported by Biju et al. (2011). In order to nullify the influence of body size, PCA were performed using nine size-corrected morphometric parameters taken from adult males. Size correction was obtained by expressing the measurements of head characters (HW, SL and EL) as percent of head length (HL) and other body measurements (HL, FAL, HAL, TL, SHL and FOL) as percent of snout to vent length (SVL). Sets of nine predictor variables were generated from PCA and all PCA factor scores were used as input variables for DFA to determine the classification success of our samples. PCA were performed using the statistical software Statistica v7.1 (StatSoft Inc.), and DFA were performed both in Statistica v7.1 (StatSoft Inc.) and XLSTAT (Addinsoft).

### Bioacoustics study

Advertisement calls of a single male were recorded for seven *Nyctibatrachus* species between 18:00–23:00 h during the years 2013–2016. Calls were recorded using a solid-state digital recorder (Marantz PMD620, 44.1 kHz sampling rate, 16-bit resolution) and a unidirectional microphone (Sennheiser ME 66) equipped with a foam windscreen. At the time of recording, the microphone was positioned at an approximate distance of 75–100 cm from the calling male and calls were monitored in real time using headphones (Sony MDR-V500). Gain level of the recorder was adjusted before each recording and the settings were maintained until the end of the recording. One recorded individual from each population was collected for molecular and morphological verification of species identity. Dry bulb and wet bulb air temperatures at the animal’s calling site were recorded to the nearest 0.1 °C using a thermometer (Jennson Delux).

We measured a total of five temporal properties that included call duration, call rise time, call fall time, number of pulses per call and pulse rate, using Raven Pro 1.4 (Charif, Waack & Strickman, 2010) of a single call for each species. Number of pulses and pulse rate were not measured for species with non-pulsatile temporal structure. One spectral property, i.e., dominant frequency, was also measured after averaging spectrum over the entire call. For better representation, spectrogram figures were prepared by using either one second time frame (for calls longer than 0.1 s) or 0.1 s time frame of the call. Terminologies and graphical representation of call properties analysed in this study follow Bee, Suyesh & Biju (2013a) and Bee, Suyesh & Biju (2013b).

### Nomenclatural acts

The electronic version of this article in Portable Document Format (PDF) will represent a published work according to the International Commission on Zoological Nomenclature (ICZN), and hence the new names contained in the electronic version are effectively published under that Code from the electronic edition alone. This published work and the nomenclatural acts it contains have been registered in ZooBank, the online registration system for the ICZN. The ZooBank LSIDs (Life Science Identifiers) can be resolved and the associated information viewed through any standard web browser by appending the LSID to the prefix “http://zoobank.org/”. The LSID for this publication is: urn:lsid:zoobank.org:pub:B27CCEEE-DA57-4CE6-B91F-7F799D3A8B9A. The online version of this work is archived and available from the following digital repositories: PeerJ, PubMed Central and CLOCKSS.

## Results

### DNA barcoding for rapid genetic identification

The resulting NJ tree showed 36 nodes corresponding to 28 known *Nyctibatrachus* species, seven candidate species (Fig. S1), and the outgroup taxon. All the ‘candidate species’ (Vences & Wake, 2007) were found to be at least 3% divergent from their closest known relatives (see Table S2 for pairwise comparisons of each species). Since uncorrected pairwise genetic divergences of minimum 3% for the mitochondrial 16S rRNA gene are known to be reliable for preliminary delimitation of amphibians species (e.g., Fouquet et al., 2007; Vieites et al., 2009; Biju et al., 2014a; Biju et al., 2014b), we considered the seven new populations of *Nyctibatrachus* frogs sampled by us as potentially new species and conducted further morphological studies for confirmation. For phylogenetic results see the ‘Genetic relationships’ section.

### Morphometric differentiation

Based on morphological similarities of the candidate species, three separate principle component analyses (PCA) and discriminant function analyses (DFA) were performed for: (1) Seven species of miniature frogs, including *Nyctibatrachus anamallaiensis*, *N. beddomii*, *N. minimus*, *N. manalari* sp. nov., *N. pulivijayani* sp. nov., *N. robinmoorei* sp. nov. and *N. sabarimalai* sp. nov.; (2) Five species of medium-sized frogs, *N. athirappillyensis* sp. nov., *N. deccanensis*, *N. kempholeyensis*, *N. minor* and *N. webilla* sp. nov.; and (3) Seven species of large-sized frogs, including *N. acanthodermis*, *N. gavi*, *N. grandis*, *N. indraneili*, *N. major*, *N. radcliffei* sp. nov. and *N. sylvaticus*. The PCA factor loadings representing the composition of PCA factors, and the parameters correlated with each PCA factor are shown in Tables S3A–S3C. The coefficients of canonical discriminant function representing the composition of canonical discriminant scores are shown in Tables S4A–S4C.

**Figure 1:**
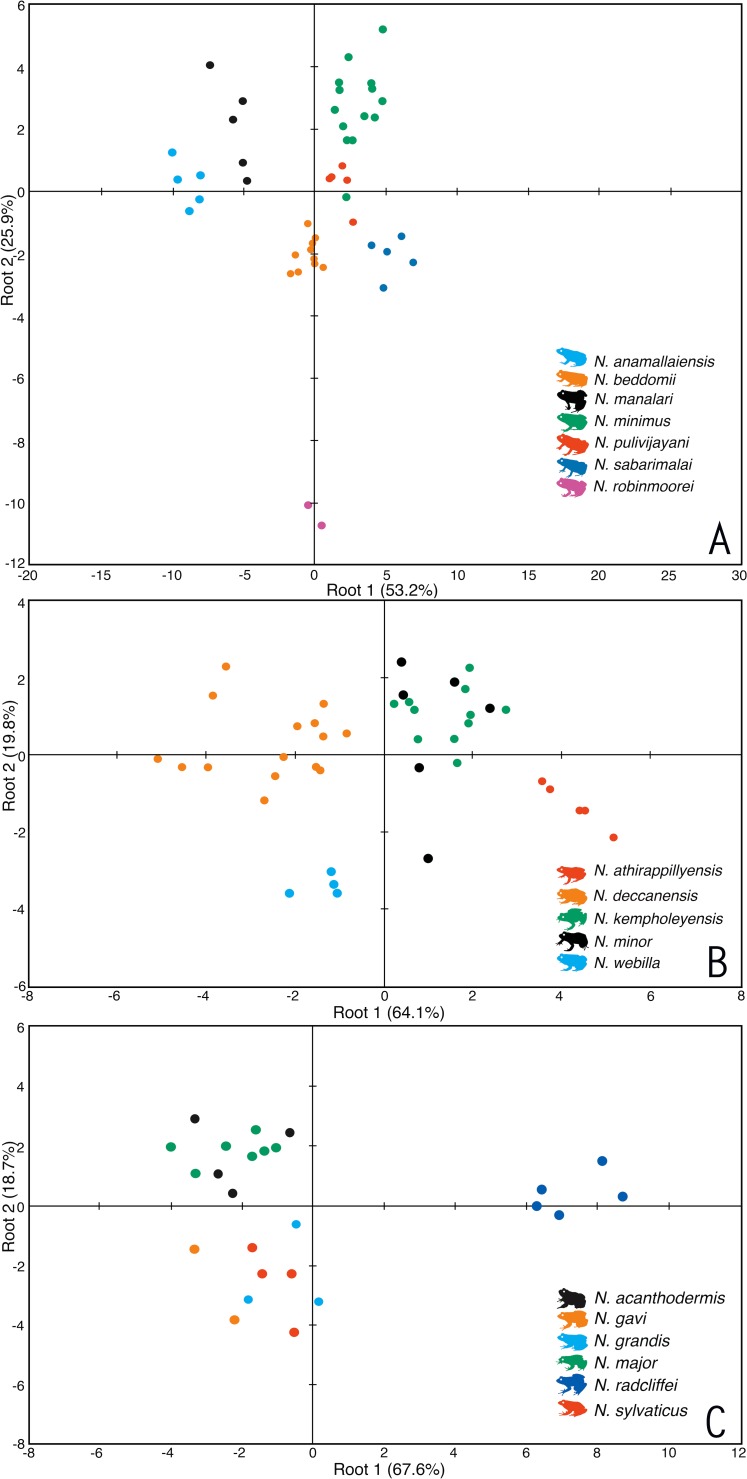
Morphometric differentiation of species using the discriminant function analyses. (A) Projection on factor plane first and second explaining 79.1% of the variations among seven miniature-sized species. (B) Projection on factor plane first and second explaining 83.9% of the variations among five medium-sized species. (C) Projection on factor plane first and second explaining 86.3% of the variations among six large-sized species.

For the miniature frogs, the first four PCA factors with eigenvalues more that 1.0 explained 78.7% of variation among the species (Table S3A). The DFA using all the PCA factors as input resulted in 100% males being classified into their respective species (Table S5A). The first five discriminant functions with eigenvalues greater than 1.0 explained 99.7% of variation among these species (Table S4A), and all the seven species of miniaturized Night Frogs formed considerably distinct clusters on the factor plane using the first two DFA roots (Fig. 1).

For the medium-sized species, the first three PCA factors had eigenvalues more that 1.0 and they explained 72.0% of variation (Table S3B). The DFA was performed using all PCA factors as input and the first three discriminant functions with eigenvalues greater than 1.0 explained 99.4% of variation among these species (Table S4B). The DFA resulted in 87.8% males being classified into their respective species (Table S5B). All the samples of *Nyctibatrachus athirappillyensis, N. deccanensis* and *N. webilla* resulted in 100% classification success and formed distinct clusters on the factor plane using the first two DFA roots (Fig. 1; Table S5B). Classification error was seen between samples of two previously known species—*N. kempholeyensis* and *N. minor*, and one sample of *N. minor* was misclassified as *N. webilla* (Table S5B). However, *N. kempholeyensis* and *N. minor* are phylogenetically and morphologically distinct from each other (Biju et al., 2011; Van Bocxlaer et al., 2012). For detailed comparison between *N. minor* and *N. webilla,* see ‘morphological comparison’ section under formal description of the latter, the ‘genetic relationships’ section and Table S2.

For the large-sized species, 77.8% of variation was explained by the first three PCA factors with eigenvalues more that 1.0 (Table S3C). The DFA was performed using all PCA factors as input but one species *Nyctibatrachus indraneili* was excluded from the analysis due to availability of a single representative. The first four discriminant functions had eigenvalues greater than 1.0 and explained 99.7% of variation among these species (Table S4C). Classification of males into their respective species was achieved successfully (100%) and all the species formed distinct clusters on the factor plane using the first two DFA roots (Fig. 1; Table S5C).

Overall, the results of DFA showed high classification success (87.8–100%), clearly indicating that the size-corrected morphological variables were useful in differentiation of all the new species from their close relatives.

### Description of new species

Our detailed morphological study supports the recognition of seven new *Nyctibatrachus* species, which can be reliably differentiated from their relatives on the basis of skin texture, dorsal folds and markings, skin colour, body shape and size, finger and toe tip morphology, and degree of webbing. These taxa are formally described below.

**Table:** 

**Figure 2:**
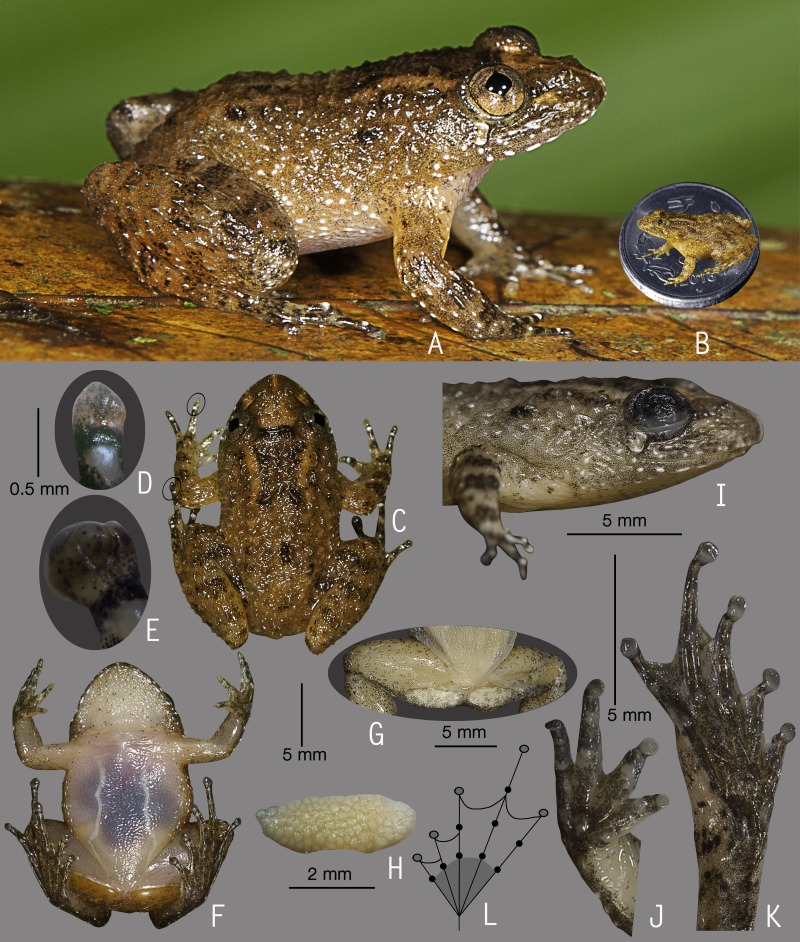
Holotype of *Nyctibatrachus athirappillyensis* sp. nov. (ZSI/WGRC/V/A/891). (A) Dorsolateral view, in life. (B) Size (SVL 20.9 mm) in comparison to the Indian five-rupee coin (24 mm diameter). (C) Dorsal view, in life. (D) Dorsal surface of third finger disc, in preservation. (E) Dorsal surface of fourth toe disc, in preservation. (F) Ventral view, in life. (G) Femoral glands, in preservation. (H) Close-up of femoral glands after removal of skin showing multiple glands. (I) Lateral view of head, in preservation. (J) Ventral view of hand, in preservation. (K) Ventral view of foot, in preservation. (L) Schematic illustration of foot webbing.

**Figure 3:**
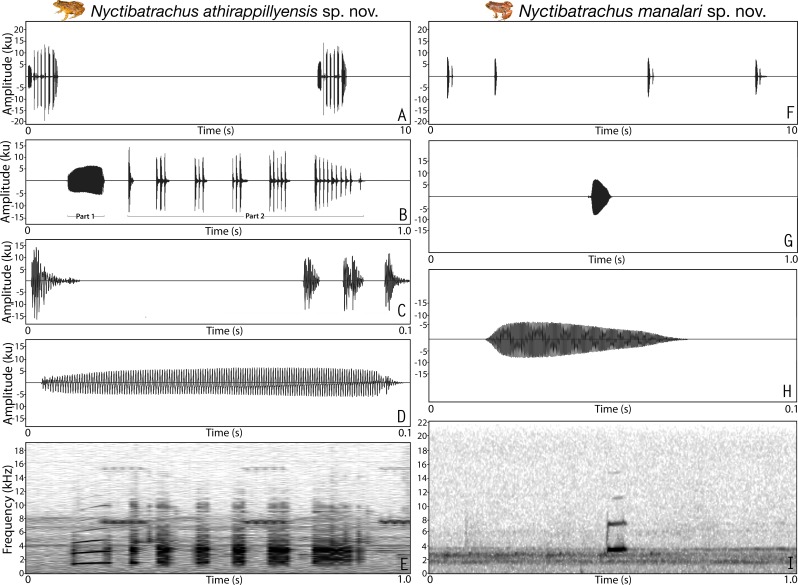
Male advertisement calls of *Nyctibatrachus athirappillyensis* sp. nov. and *Nyctibatrachus manalari* sp. nov. (A–E) *Nyctibatrachus athirappillyensis* sp. nov. (A) 10 s segment. (B) 1 s segment showing part 1 (non-pulsatile) and part 2 (pulsatile) of a single call. (C) 0.1 s segment showing the part 2 of a single call. (D) 0.1 s segment showing the part 1 of a single call. (E) Spectrogram of 1 s call segment. (F–I) *Nyctibatrachus manalari* sp. nov. (F) 10 s segment. (G) 1 s segment. (H) 0.1 s segment showing a single non-pulsatile call. (I) Spectrogram of 1 s call segment.

***Nyctibatrachus athirappillyensis*** sp. nov.
urn:lsid:zoobank.org:act:4756A24F-60E1-4B85-956F-5FC3CE6B876F
Athirappilly Night Frog
(Figs. 1–3; Tables S1–S8)

**Holotype.** ZSI/WGRC/V/A/891, adult male, from Thavalakuzhipara (10°16′53″N 76°41′25.6″E, 530 m), Vazhachal forest division, Thrissur district, Kerala state, India, collected by SDB and SG on 11 September 2015.

**Paratypes.** ZSI/WGRC/V/A/892–895, four adult males, and ZSI/WGRC/V/A/896, adult female, collected from the same locality as holotype, by SDB and SG on 11 July 2016.

**Etymology.** The species epithet is an adjective that refers to Athirappilly falls, which is in close vicinity of the type locality.

**Diagnosis.** *Nyctibatrachus athirappillyensis* can be distinguished from known congeners by the following combination of morphological characters: (1) small male adult size (SVL 20.9–22.8 mm, *N* = 5); (2) head width nearly equal to head length (male HW/HL ratio 96.3–100%, *N* = 5); (3) a well developed ridge extending from the lip over the tip of the snout to between the nostrils, at which point it bifurcates, producing an inverted ‘Y’; (4) third finger disc slightly wider than finger width (male FD_III_ 0.5–0.7, FW_III_ 0.3–0.4, *N* = 5), with dorso-terminal groove and cover rounded distally; (5) fourth toe disc moderately wider than toe width (male TD_IV_ 0.7–0.8, TW_IV_ 0.3–0.5, *N* = 5), with dorso-terminal groove and cover notched distally; (6) presence of two palmar tubercles; (7) foot webbing moderately large, fourth toe webbing nearly extending up to the first subarticular tubercle on either side; (8) thigh nearly equal to shank (male TL/SHL ratio 98–100%, *N* = 5); and (9) shank nearly equal to foot length (male SHL/FOL ratio 98.1–102%, *N* = 5).

**Morphological comparison.** *Nyctibatrachus athirappillyensis* can be distinguished from its congeners *N. acanthodermis*, *N. danieli, N. dattatreyaensis, N. gavi, N. grandis, N. humayuni, N. indraneili, N. jog, N. karnatakaensis, N. kumbara, N. major, N. periyar, N. petraeus, N. poocha, N. radcliffei* sp. nov., *N. sanctipalustris, N. sylvaticus* and *N. vrijeuni* by its relatively smaller snout-vent size, male SVL 20.9–22.8 mm (vs. larger, male SVL 24.2–76.9 mm) and dorsal skin less prominently wrinkled and/or granular (vs. prominently wrinkled and/or granular). *Nyctibatrachus athirappillyensis* differs from *N. aliciae, N. deveni, N. pillaii, N. shiradi* and *N. vasanthi* by its dorsal skin less prominently wrinkled and/or granular (vs. prominently wrinkled and/or granular in all five species), and third finger disc with dorso-terminal groove and cover rounded distally (vs. third finger disc with dorso-terminal groove and cover notched distally in *N. aliciae*, *N. deveni* and *N. shiradi*; third finger disc with dorso-terminal groove and cover bifurcate distally in *N. pillaii* and *N. vasanthi*). *Nyctibatrachus athirappillyensis* differs from *N. anamallaiensis, N. beddomii, N. manalari* sp. nov., *N. minimus, N. pulivijayani* sp. nov., *N. robinmoorei* sp. nov. and *N. sabarimalai* sp. nov. by its relatively larger male snout-vent size, male SVL 20.9–22.8 mm (vs. smaller, male SVL 10.0–18.0 mm) and presence of webbing between toes (vs. absent).

Because of the comparable snout-vent size, *Nyctibatrachus athirappillyensis* could be confused with the previously known species *N. deccanensis*, *N. kempholeyensis* and *N. minor*, and the new species *N. webilla* sp. nov. However, *N. athirappillyensis* differs from *N. deccanensis* by its relatively large snout-vent size, male SVL 20.9–22.8, *N* = 5 (vs. male SVL 16.1–20.8, *N* = 15), head width subequal or equal to head length, male HW/HL ratio 96.3–100%, *N* = 5 (vs. head wider than long, male HW/HL ratio 101.3–125.8%, *N* = 15), snout length relatively larger than head length, male SL/HL ratio of 41.8–48.1%, *N* = 5 (vs. male SL/HL ratio of 30.0–38.7%, *N* = 15), third finger disc with dorso-terminal groove and cover rounded distally (vs. without groove), fourth toe disc with dorso-terminal groove and cover notched distally (vs. with dorso-terminal groove and cover bifurcate distally), fourth toe webbing nearly extending up to the first subarticular tubercle on either side, I1–1^3^/_4_II1–2^+^III1–2^+^IV2^+^–1V (vs. up to the second subarticular tubercle on either side), and flesh or off white ventral coloration in life (vs. red, reddish-orange or reddish-brown).

*Nyctibatrachus athirappillyensis* differs from *N. kempholeyensis* by its snout vertical in lateral view (vs. rounded), presence of two palmar tubercles (vs. single), and fourth toe webbing nearly extending up to the first subarticular tubercle on either side, I1–1^3^/_4_II1–2^+^III1–2^+^IV2^+^–1V (vs. below, I1–2^−^II1–2^1^/_4_III1–2^1^/_2_IV2^1^/_2_–1V).

*Nyctibatrachus athirappillyensis* differs from *N. minor* by its relatively larger snout-vent size, male SVL 20.9–22.8 mm, *N* = 5 (vs. male SVL 15.4–17.9 mm, *N* = 6), third finger disc with dorso-terminal groove and cover rounded distally (vs. with dorso-terminal groove and cover bifurcate distally), fourth toe disc with dorso-terminal groove and cover notched distally (vs. with dorso-terminal groove and cover bifurcate distally), relatively shorter forearm length than hand length, male FAL/HAL ratio 65.5–69.8%, *N* = 5 (vs. male FAL/HAL ratio 72.1–78.9%, *N* = 6), and foot webbing large (vs. absence of webbing).

*Nyctibatrachus athirappillyensis* differs from *N*. *webilla* by its relatively larger snout-vent size, male SVL 20.9–22.8, *N* = 5 (vs. male SVL 18.7–20.7, *N* = 4), head width subequal or equal to head length, male HW/HL ratio 96.3–100%, *N* = 5 (vs. head wider than long, male HW/HL ratio 110.8–117.4%, *N* = 4), third finger disc with dorso-terminal groove and cover rounded distally (vs. without groove), fourth toe disc with dorso-terminal groove and cover notched distally (vs. with dorso-terminal groove and cover bifurcate distally), thigh nearly equal to shank, male TL/SHL ratio 98–100%, *N* = 5 (vs. longer, male TL/SHL ratio 106.9–111.1%, *N* = 4), shank nearly equal to foot length, male SHL/FOL ratio 98.1–102%, *N* = 5 (vs. shorter, male SHL/FOL ratio 90.0–93.5%, *N* = 4), fourth toe webbing nearly extending up to the first subarticular tubercle on either side, I1–1^3^/_4_II1–2^+^III1–2^+^IV2^+^–1V (vs. just above the third subarticular tubercle, I2^−^–2^−^II2^−^–3III3^−^–4^−^IV4^−^–3^−^V), and flesh or off white ventral coloration in life (vs. red, reddish-orange or reddish-brown).

**Description of holotype** ***(measurements in mm)***. Adult male (SVL 20.9); head width subequal to its length (HW 7.6, HL 7.7); snout nearly rounded in dorsal view, vertical in lateral view, its length (SL 3.7) longer than horizontal diameter of the eye (EL 2.4); loreal region obtuse with indistinct canthus rostralis; interorbital space flat, wider (IUE 2.5) than the upper eyelid (UEW 1.2) and internarial distance (IN 1.9); nostril closer to eye (EN 1.4) than the tip of snout (NS 1.7); tympanum indistinct; vomerine ridge present, bearing small teeth, with an angle of 45° to body axis, closer to each other than choanae, longer than the distance between them; tongue moderately large, emarginated, bearing no median lingual process; supratympanic fold weakly developed. Forearm (FAL 3.6) shorter than hand length (HAL 5.4), fingers with dermal fringes, finger length formula: I < II < IV < III, finger discs slightly wide compared to finger width (FD_I_ 0.5, FW_I_ 0.4; FD_II_ 0.5, FW_II_ 0.3; FD_III_ 0.5, FW_III_ 0.4; FD_IV_ 0.5, FW_IV_ 0.4), finger disc with dorso-terminal groove, cover rounded distally; subarticular tubercles prominent, oval, single, all present; prepollex distinct, oval; two palmar tubercles, distinct, oval; nuptial pads present. Thigh length (TL 10.0) subequal to shank (SHL 10.2), and equal to foot (FOL 10.0), relative toe lengths I < II < V < III < IV, toe discs moderately wide compared to toe width (TD_I_ 0.7, TW_I_ 0.4; TD_II_ 0.8, TW_II_ 0.4; TD_III_ 0.9, TW_III_ 0.5; TD_IV_ 0.8, TW_IV_ 0.5; TD_V_ 0.6, TW_V_ 0.5), toe disc with dorso-terminal groove, cover notched distally; foot webbing moderately large: I1–1^3^/_4_II1–2^+^III1–2^+^IV2^+^–1V, fourth toe webbing nearly extending up to the first subarticular tubercle on either side (Figs. 2K and 2L); subarticular tubercles well developed, oval, single, all present; inner metatarsal tubercle present, oval; outer metatarsal tubercle absent; dermal fringe along toes I and V present, weakly developed.

Skin of snout shagreened to granular, upper eyelids tuberculate, sides of head, anterior and posterior parts of back and upper and lower parts of flank weakly wrinkled with spinular projections; subocular gland prominent, extending from the posterior ventral border of the orbit towards the posterior axis of the mandibles; dorsal surfaces of forelimb, thigh and shank with weakly developed folds or wrinkles and spinular projections; a well developed ridge extending from the lip over the tip of the snout to between the nostrils, at which point it bifurcates, producing an inverted ‘Y’; a well developed glandular fold between the eyes; ventral surface of throat with weakly developed longitudinal folds, chest shagreened, prominent glandular projections on the margin of lower jaw, belly and limbs shagreened; a pair of prominent femoral glands present on the ventral surface of thighs (Figs. 2F–2H).

**Colour of holotype.** *In life.* Dorsal and lateral side of head greyish-brown with scattered black spots, upper eyelids dark brown (Figs. 2A and 2C); a dark brown stripe between the eyes demarcating a triangular light orangish-brown patch on the snout, a pair of light orangish-brown longitudinal dorsal bands starting from behind the eyelids and extending up to the middle of dorsum (Fig. 2C); forelimbs (including fingers) and hind limbs (including toes) light orangish-brown with dark brown transverse bands; white glandular projections on the throat margins, lateral surfaces of the head and abdomen, margins of limbs, fingers and toes; webbing grey with dark grey spots; femoral glands orangish-brown with brown spots (Fig. 2F). *In preservation*. Dorsum grey with scattered dark grey spots, a pair of light greyish-brown longitudinal dorsal bands starting from behind the eyelids and extending up to the middle of dorsum; limbs, fingers and toes light grey with dark grey cross-bands; ventral surfaces greyish-white with scattered dark grey spots on the throat, forelimbs and hindlimbs, margins of limbs darker in colour.

**Variations.** Morphometric data from five adult males and an adult female, including the holotype, is given in Table S6. Overall, the colour and meristic characters of the paratypes are similar to the holotype. *Colour in preservation*. ZSI/WGRC/V/A/892: dorsum darker grey with more prominent dark grey spots; ZSI/WGRC/V/A/893–895: dorsum uniformly darker grey, ventral surfaces of throat, forelimbs and hindlimbs with more prominent dark grey spots.

**Secondary sexual characters.** *Male*, femoral glands present, nuptial pad on finger I present. *Female* (ZSI/WGRC/V/A/896), small pigmented eggs present (diameter 1.1  ± 0.4mm, *N* = 10).

**Vocalization.** Male (ZSI/WGRC/V/A/893) of *Nyctibatrachus athirappillyensis* produced a single type of call with two distinct parts, hereafter termed part 1 and part 2. Calls were not delivered in groups and the call parts had a fixed order, i.e., part 1 followed by part 2. The duration of the entire call was 755.7 ms and had a dominant frequency of 3.4 kHz. Part 1 had a single pulse (non-pulsatile) and was 93.5 ms in duration. Part 1 had a rise time of 43.6 ms and was without any significant fall time (7.3 ms). The overall dominant frequency of part 1 was 1.6 kHz with three distinct frequency peaks. On the other hand, part 2 had a pulsatile temporal structure and was much longer in duration (595.8 ms) compared to part 1, with 25 unevenly spaced pulses delivered at a rate of 42.2 pulses/s. The amplitude envelope of part 2 was characterized by a short rise time of 1.7 ms, fall time of 593.5 ms, and the overall dominant frequency of 3.4 kHz with two distinct frequency peaks (Table S8; Figs. 3A–3E). Air temperature at the time of recording: dry bulb 25 °C, wet bulb 24 °C.

**Distribution and natural history.** *Nyctibatrachus athirappillyensis* is currently known only from its type locality in the southern Western Ghats state of Kerala. All the specimens were collected from shallow streams or marshy areas covered with thick vegetation or leaf litter. Collection site was located inside a secondary forest. Calling males were found hiding under vegetation either inside the shallow stream or on the edges. Calls were heard and recorded during the late evening between 18:00–22:00 h.

**Remark.** Biju et al. (2011) erroneously interpreted the “fourth toe disc with dorso-terminal groove, cover rounded distally” in *Nyctibatrachus kempholeyensis*. In the present study we confirm that the fourth toe disc of *N. kempholeyensis* has a dorso-terminal groove with cover notched distally.

**Table:** 

**Figure 4:**
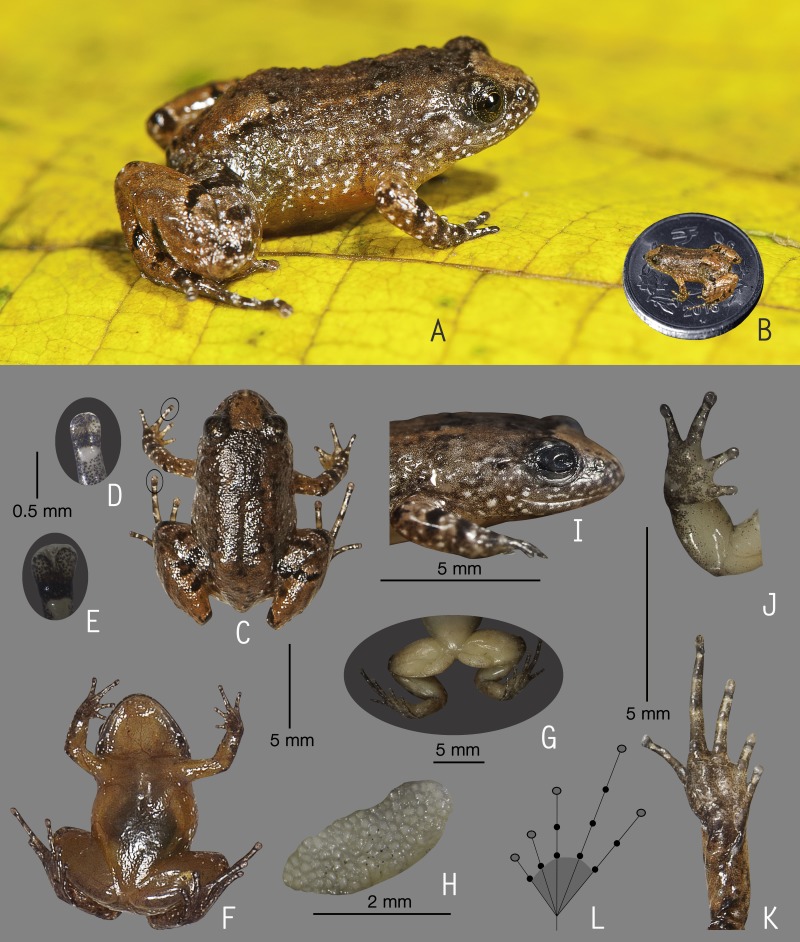
Holotype of *Nyctibatrachus manalari* sp. nov. (ZSI/WGRC/V/A/897). (A) Dorsolateral view, in life. (B) Size (SVL 13.8 mm) in comparison to the Indian five-rupee coin (24 mm diameter). (C) Dorsal view, in life. (D) Dorsal surface of third finger disc, in preservation. (E) Dorsal surface of fourth toe disc, in preservation. (F) Ventral view, in life. (G) Femoral glands, in preservation. (H) Close-up of femoral glands after removal of skin showing multiple glands. (I) Lateral view of head, in preservation. (J) Ventral view of hand, in preservation. (K) Ventral view of foot, in preservation. (L) Schematic illustration of foot webbing.

***Nyctibatrachus manalari*** sp. nov.
urn:lsid:zoobank.org:act:9B10F3DA-07E4-4A78-B96B-C76C3C1578F6
Manalar Night Frog
(Figs. 1–4; Tables S1–S8)

**Holotype.** ZSI/WGRC/V/A/897, adult male, from Upper Manalar (09°34′29.31″N 77° 20′10.27″E, 1564 m), Periyar Tiger Reserve, Idukki district, Kerala state, India, collected by SDB and SG on 15 July 2016.

**Paratypes.** ZSI/WGRC/V/A/898–901, four adult males, collected along with the holotype.

**Other material studied.** SDBDU 2015.2994, from the same locality as the type series, collected by SDB and SG on 05 July 2015, was used only for genetic identification of species.

**Etymology.** The species is named after the type locality Upper Manalar in Periyar Tiger Reserve, from where the type series was collected. The specific name *manalari* is a noun in the genitive case.

**Diagnosis.** *Nyctibatrachus manalari* can be distinguished from known congeners by the following combination of morphological characters: (1) miniature-sized adult males (SVL 13.1–15.4 mm, *N* = 5); (2) head wider than long (male HW/HL ratio 115.7–135.6%, *N* = 5); (3) presence of weakly developed dorsolateral glandular folds separated by scattered granular projections; (4) third finger disc slightly wider than finger width (male FD_III_ 0.3–0.4, FW_III_ 0.2–0.3, *N* = 5), with dorso-terminal groove and cover bifurcate distally; (5) fourth toe disc slightly wider than toe width (male TD_IV_ 0.4–0.6, TW_IV_ 0.2–0.3, *N* = 5), with dorso-terminal groove, cover bifurcate distally; (6) presence of single palmar tubercle; (7) foot webbing absent; (8) thigh nearly equal to shank length (male TL/SHL ratio 98.4–101.5%, *N* = 5); (9) thigh shorter than foot length (male TL/FOL ratio 84.7–90.7%, *N* = 5); (10) shank shorter than foot length (male SHL/FOL ratio 86.1–90.7%, *N* = 5); and (11) light reddish-brown ventral coloration in life.

**Morphological comparison.** *Nyctibatrachus manalari* species can be distinguished from all its congeners, expect *N. anamallaiensis, N. athirappillyensis* sp. nov., *N. beddomii, N. deccanensis*, *N. kempholeyensis, N. minimus, N. minor, N. pulivijayani* sp. nov., *N. robinmoorei* sp. nov., *N. sabarimalai* sp. nov. and *N. webilla* sp. nov., by its smaller snout-vent size, male SVL 13.1–15.4 mm (vs. larger, male SVL 18.1–76.9 mm), absence of webbing between toes (vs. present), and dorsal skin not prominently wrinkled and/or granular (vs. prominently wrinkled and/or granular). *Nyctibatrachus manalari* differs from *N. athirappillyensis, N. deccanensis* and *N. kempholeyensis* by absence of webbing between toes (vs. present); differs from *N. minor* by its dorsal skin without distinct dorsolateral glandular folds separated by an ‘X’ pattern on anterior half of back (vs. present); and differs from *N. webilla* by its third finger disc with dorso-terminal groove and cover bifurcate distally (vs. third finger without dorso-terminal groove).

Because of the comparable snout-vent size, *Nyctibatrachus manalari* could be confused with the previously known species *N. anamallaiensis*, *N. beddomii* and *N. minimus*, and three new species *N. pulivijayani*, *N. robinmoorei* and *N. sabarimalai*. However, *N. manalari* differs from *N. anamallaiensis* by its relatively larger male SL/HL ratio of 41.2–50%, *N* = 5 (vs. male SL/HL ratio of 29.4–34.9%, *N* = 5), eye length shorter than snout length, male EL/SL ratio 66.7–94.4%, *N* = 5 (vs. longer, male EL/SL ratio 125–140%, *N* = 5), fingers with dermal fringe (vs. absent), and light reddish-brown ventral coloration in life (vs. flesh or off white).

*Nyctibatrachus manalari* differs from *N. beddomii* by its dorsolateral glandular folds separated by scattered granular projections (vs. dorsal skin finely granular), forearm considerably shorter than hand length, male FAL/HAL ratio 58.8–73.5%, *N* = 5 (vs. nearly equal, male FAL/HAL ratio 93.3–103.7%, *N* = 10), relatively smaller male TL/FOL ratio of 84.7–90.7%, *N* = 5 (vs. male TL/FOL ratio of 91.4–107.8%, *N* = 10) and relatively smaller male SHL/FOL ratio of 86.1–90.7%, *N* = 5 (vs. male SHL/FOL ratio of 91.4–111.3%, *N* = 10).

*Nyctibatrachus manalari* differs from *N. minimus* by its dorsolateral glandular folds separated by scattered granular projections (vs. dorsal skin having faint and interrupted glandular folds), relatively smaller male HL/SVL ratio of 30.4–34.9%, *N* = 5 (vs. male HL/SVL ratio of 37.8–44.6%, *N* = 14), relatively smaller male TL/FOL ratio of 84.7–90.7%, *N* = 5 (vs. male TL/FOL ratio of 91.2–121.6%, *N* = 14) and relatively smaller male SHL/FOL ratio of 86.1–90.7%, *N* = 5 (vs. male SHL/FOL ratio of 97.1–125.5%, *N* = 14).

*Nyctibatrachus manalari* differs from *N. pulivijayani* by its head wider than long, male HW/HL ratio 115.7–135.6%, *N* = 5 (vs. nearly equal, male HW/HL ratio 98.3–102%, *N* = 5), relatively smaller male HL/SVL ratio of 30.4–34.9%, *N* = 5 (vs. male HL/SVL ratio 35.9–39.5%, *N* = 5), relatively larger male EL/HL ratio of 33.3–39.5%, *N* = 5 (vs. male EL/HL ratio of 25.9–32.7%, *N* = 5), thigh shorter than foot, male TL/FOL ratio 84.7–90.7%, *N* = 5 (vs. nearly equal, male TL/FOL ratio 95.5–106%, *N* = 5), shank shorter than foot, male SHL/FOL ratio 86.1–90.7%, *N* = 5 (vs. nearly equal, male SHL/FOL ratio 98.5–103%, *N* = 5), fingers with dermal fringe (vs. absent), and light reddish-brown ventral coloration in life (vs. flesh or off white).

*Nyctibatrachus manalari* differs from *N. robinmoorei* by its head wider than long, male HW/HL ratio 115.7–135.6%, *N* = 5 (vs. nearly equal, male HW/HL ratio 97.6–100%, *N* = 2), forearm shorter than hand length, male FAL/HAL ratio 58.8–73.5%, *N* = 5 (vs. longer, male FAL/HAL ratio 105.3–111.8%, *N* = 2), thigh nearly equal to shank, male TL/SHL ratio 98.4–101.5%, *N* = 5 (vs. longer, male TL/SHL ratio 127.5–130.8%, *N* = 2), thigh shorter than foot, male TL/FOL ratio 84.7–90.7%, *N* = 5 (vs. longer, male TL/FOL ratio 122.6–130.8%, *N* = 2), shank shorter than foot, male SHL/FOL ratio 86.1–90.7%, *N* = 5 (vs. nearly equal, male SHL/FOL ratio 96.2–100%, *N* = 2), fingers with dermal fringe (vs. absent), and light reddish-brown ventral coloration in life (vs. flesh or off white).

*Nyctibatrachus manalari* differs from *N. sabarimalai* by its head wider than long, male HW/HL ratio 115.7–135.6%, *N* = 5 (vs. head longer than wide, male HW/HL ratio 82.4–89.8%, *N* = 5), relatively smaller male HL/SVL ratio of 30.4–34.9%, *N* = 5 (vs. male HL/SVL ratio 38.3–41.5%, *N* = 5), relatively larger male EL/HL ratio of 33.3–39.5%, *N* = 5 (vs. male EL/HL ratio of 27.5–30.2%, *N* = 5), relatively smaller male FAL/HAL ratio of 58.8–73.5%, *N* = 5 (vs. male FAL/HAL ratio of 85.7–96.6%, *N* = 5), thigh shorter than foot, male TL/FOL ratio 84.7–90.7%, *N* = 5 (vs. nearly equal, male TL/FOL ratio 96.9–101.6%, *N* = 5), shank shorter than foot, male SHL/FOL ratio 86.1–90.7%, *N* = 5 (vs. nearly equal, male SHL/FOL ratio 98.4–100%, *N* = 5), fingers with dermal fringe (vs. absent), and light reddish-brown ventral coloration in life (vs. flesh or off white).

**Description of holotype *(measurements in mm)*.** Adult male (SVL 13.8); head small, wider than long (HW 5.0, HL 4.2); snout rounded in dorsal and lateral view, its length (SL 1.9) longer than horizontal diameter of eye (EL 1.4); loreal region acute with indistinct canthus rostralis; interorbital space flat, wider (IUE 1.9) than upper eyelid (UEW 0.7) and internarial distance (IN 1.5); nostril closer to eye (EN 0.6) than the tip of snout (NS 0.9); tympanum indistinct; vomerine ridge weakly developed, bearing a few small teeth, at an angle of 70° to the body axis, closer to each other than choanae, shorter than the distance between them; tongue emarginated, bearing no medial lingual process. Forearm (FAL 2.0) shorter than hand length (HAL 3.4), finger length formula: I <II < IV < III, finger discs slightly wider compared to its width (FD_I_ 0.2, FW_I_ 0.1; FD_II_ 0.3, FW_II_ 0.2; FD_III_ 0.3, FW_III_ 0.2; FD_IV_ 0.3, FW_IV_ 0.2), finger disc with dorso-terminal groove, cover bifurcate distally; subarticular tubercles prominent, oval, single, all present; prepollex distinct, oval; single palmar tubercle, oval, distinct; nuptial pads present. Thigh length (TL 6.1) subequal to shank (SHL 6.2), and shorter than foot (FOL 7.2), relative toe lengths I < II < V < III < IV, toe discs slightly wider compared to toe width (TD_I_ 0.4, TW_I_ 0.3; TD_II_ 0.4, TW_II_ 0.3; TD_III_ 0.4, TW_III_ 0.3; TD_IV_ 0.5, TW_IV_ 0.3; TD_V_ 0.4, TW_V_ 0.3), toe disc with dorso-terminal groove, cover bifurcate distally; foot webbing absent; subarticular tubercles well developed, oval, single, all present; inner metatarsal tubercle present, oval; outer metatarsal tubercle weakly developed.

Skin of snout shagreened, upper eyelids with a few prominent glandular warts especially on the margins, presence of weakly developed dorsolateral glandular folds separated by scattered granular projections; sides of head, anterior and posterior parts of back, and upper and lower parts of flank shagreened with scattered glandular projections; subocular gland indistinct; upper surface of arms and legs shagreened with weakly developed granular projections; ventral surfaces smooth; a pair of prominent femoral glands present on the ventral surface of thighs (Figs. 4G and 4H).

**Colour of holotype.** *In life*. Dorsum reddish-brown, with a pair of faint orangish-brown longitudinal bands starting from behind the eyelids and extending up to the middle of dorsum, another orangish-brown stripe between the eyes demarcating a triangular orangish-brown patch on the snout (Fig. 4C); lateral sides of the head light greyish-brown with scattered white spots, upper eyelids dark brown; upper and lower parts of flank light orangish-brown with scattered minute white spots; forelimbs (including fingers) and hind limbs (including toes) light reddish-brown with dark brown transverse bands and scattered white spots. Ventral surfaces light reddish-brown in life (Fig. 4F), hand and foot lighter in colour than the chest and abdomen. *In preservation*. Dorsum and upper eyelids dark greyish-brown, with a pair of faint light brown longitudinal bands starting from behind the eyelids and extending upto the middle of dorsum, another light brown stripe between the eyes demarcating a triangular light brown patch on the snout; lateral sides of head light grey; forelimbs (including fingers) and hind limbs (including toes) light grey with dark grey transverse bands; anterior and posterior parts of flanks grey; ventral surfaces light greyish-white, hand and foot grey.

**Variations.** Morphometric data from five adult males, including the holotype, is given in Table S6. Overall, the colour and meristic characters of the paratypes are similar to the holotype. *Colour in preservation*. ZSI/WGRC/V/A/898: dorsum light brown in colour with dark grey spots; ZSI/WGRC/V/A/899 and ZSI/WGRC/V/A/901: dorsum darker grey in colour; ZSI/WGRC/V/A/900: dorsum lighter grey in colour.

**Secondary sexual characters.** *Male* (ZSI/WGRC/V/A/897), femoral glands present (Figs. 4G and 4H), nuptial pads weakly developed.

**Vocalization**. Male (ZSI/WGRC/V/A/897) of *Nyctibatrachus manalari* produced a single type of call. The calls had a single pulse and were not delivered in groups. A single call had the duration of 51.1 ms. Amplitude envelope of the call was characterized by a rise time of 6.7 ms, fall time of 34.7 ms. and the overall dominant frequency of 3.6 kHz with two broad peaks (Table S8; Figs. 3F–3I).

**Distribution and natural history.** *Nyctibatrachus manalari* is currently known only from its type locality, which is located south of Palghat gap in the Western Ghats state of Kerala. Animals were found hiding under herbs and grasses growing on or at the edges of a large rocky area inside a primary evergreen forest patch. Calling males were located and recorded at night (between 19:00–21:00 h), but calls were also heard during the day (around 14:00 h). One of the calling males was found next to an egg clutch (eight eggs) deposited under the ground vegetation.

**Table:** 

**Figure 5:**
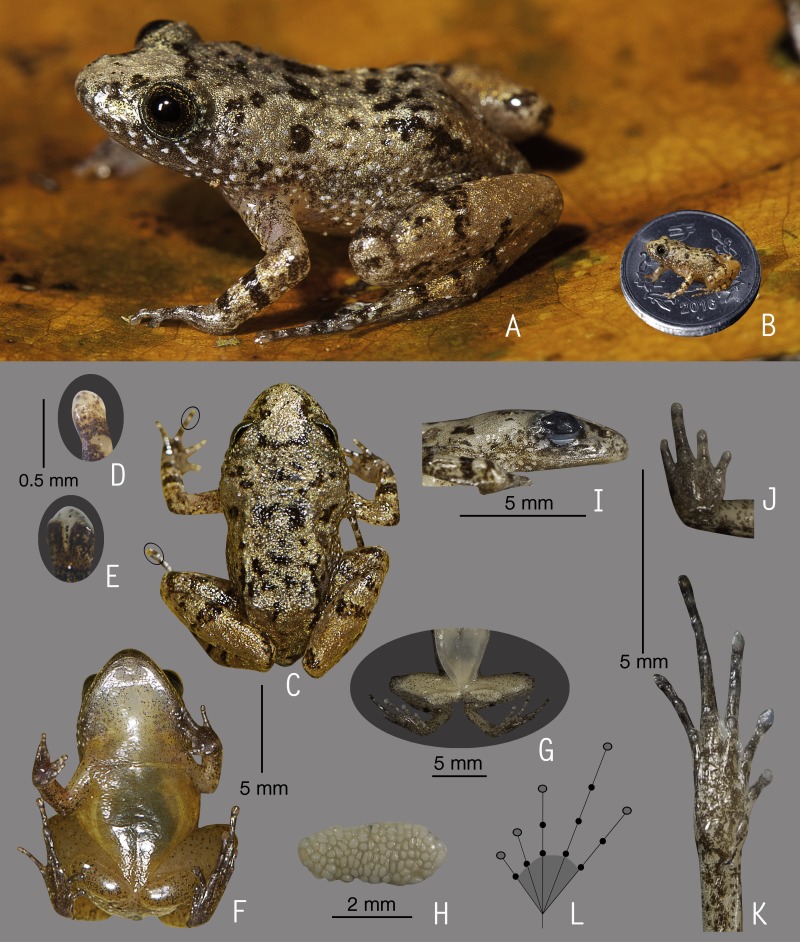
Holotype of *Nyctibatrachus pulivijayani* sp. nov. (ZSI/WGRC/V/A/902). (A) Dorsolateral view, in life. (B) Size (SVL 13.3 mm) in comparison to the Indian five-rupee coin (24 mm diameter). (C) Dorsal view, in life. (D) Dorsal surface third finger disc, in preservation. (E) Dorsal surface of fourth toe disc, in preservation. (F) Ventral view, in life. (G) Femoral glands, in preservation. (H) Close-up of femoral glands after removal of skin showing multiple glands. (I) Lateral view of head, in preservation. (J) Ventral view of hand, in preservation. (K) Ventral view of foot, in preservation. (L) Schematic illustration of foot webbing.

***Nyctibatrachus pulivijayani*** sp. nov.
urn:lsid:zoobank.org:act:E4F167C1-8340-4779-8CEF-622AC7AB350C
Vijayan’s Night Frog
(Figs. 1 and 5; Tables S1–S7)

**Holotype.** ZSI/WGRC/V/A/902, adult male, from Pandipath (08°40′42.0″N 77°11′38.6″E, 1,250 m), Thiruvananthapuram district, Kerala state, India, collected by SDB, SG and Vijayan on 19 June 2016.

**Paratypes.** ZSI/WGRC/V/A/903–905, three adult males collected along with the holotype, and ZSI/WGRC/V/A/906, adult male, collected from the same locality as holotype, by SDB and SG on 29 June 2015.

**Etymology.** This species is named after Mr. Vijayan Kani for consistently offering tremendous field support over two decades to SDB and his students during studies in the Western Ghats. Vijayan, a tribal from Agasthyamala hills of Kerala, acquired the name ‘Pulivijayan’ after he braved a leopard’s attack. The name is derived from two words–‘puli’ meaning leopard in Malayalam (official language of Kerala state) and ‘vijayan’. The species epithet ‘*pulivijayani*’ is used as a noun in the genitive case. The specific word ‘puli’ also refers to leopard-like spots observed on the dorsal surface of this species.

**Diagnosis.** *Nyctibatrachus pulivijayani* can be distinguished from known congeners by the following combination of morphological characters: (1) miniature-sized adult males (SVL 13.3–14.9 mm, *N* = 5); (2) head width nearly equal to head length (male HW/HL ratio 98.3–102%, *N* = 5); (3) presence of weakly developed dorsolateral glandular folds separated by scattered granular projections; (4) dorsum light brown to silvery-brown with prominent dark brown spots in life; (5) third finger disc slightly wider than finger width (male FD_III_ 0.3–0.4, FW_III_ 0.2–0.3, *N* = 5), with dorso-terminal groove and cover bifurcate distally; (6) fourth toe disc slightly wider than toe width (male TD_IV_ 0.4–0.5, TW_IV_ 0.2–0.3, *N* = 5), with dorso-terminal groove and cover bifurcate distally; (7) presence of single palmar tubercle; (8) foot webbing absent; (9) thigh nearly equal to shank length (male TL/SHL ratio 94–104.5%, *N* = 5); and (10) thigh nearly equal to foot length (male TL/FOL ratio 95.5–106%, *N* = 5).

**Morphological comparison.** *Nyctibatrachus pulivijayani* can be distinguished from all its congeners, expect *N. anamallaiensis, N. athirappillyensis* sp. nov., *N. beddomii, N. deccanensis*, *N. kempholeyensis, N. manalari* sp. nov., *N. minimus, N. minor, N. robinmoorei* sp. nov., *N. sabarimalai* sp. nov. and *N. webilla* sp. nov. by its smaller snout-vent size, male SVL 13.3–14.9 mm (vs. larger, male SVL 18.1–76.9 mm), dorsal skin not prominently wrinkled and/or granular (vs. prominently wrinkled and/or granular), and absence of webbing between toes (vs. present); differs from *N. athirappillyensis, N. deccanensis* and *N. kempholeyensis* by absence of webbing between toes (vs. present); differs from *N. minor* by its dorsal skin without distinct dorsolateral glandular folds separated by an ‘X’ pattern on anterior half of back (vs. present); and differs from *N. webilla* by its third finger disc with dorso-terminal groove and cover bifurcate distally (vs. third finger without dorso-terminal groove).

Because of the comparable snout-vent size, *Nyctibatrachus pulivijayani* could be confused with three previously known species *N. anamallaiensis*, *N. beddomii* and *N. minimus*, and three new species *N. manalari*, *N. robinmoorei* and *N. sabarimalai*. However, *N. pulivijayani* differs from *N. anamallaiensis* by its dorsum light brown to silvery-brown with prominent dark brown spots in life (vs. dorsum dark greyish or reddish-brown without prominent dark spots), head width nearly equal to head length, male HW/HL ratio 98.3–102%, *N* = 5 (vs. head wider than long, male HW/HL ratio 115.7–126.1%, *N* = 5), relatively larger male SL/HL ratio of 37.9–48.1%, *N* = 5 (vs. male SL/HL ratio of 29.4–34.9%, *N* = 5) and eye length shorter than snout length, male EL/SL ratio 68–78.3%, *N* = 5 (vs. longer, male EL/SL ratio 125–140%, *N* = 5).

*Nyctibatrachus pulivijayani* differs from *N. beddomii* by its dorsolateral glandular folds separated by scattered granular projections (vs. dorsal skin finely granular), head width nearly equal to head length, male HW/HL ratio 98.3–102%, *N* = 5 (vs. head wider than long, male HW/HL ratio 106.6–129.3%, *N* = 10), and forearm shorter than hand length, male FAL/HAL ratio 71.4–84.8%, *N* = 5 (vs. nearly equal, male FAL/HAL ratio 93.3–103.7%, *N* = 10).

*Nyctibatrachus pulivijayani* differs from *N. minimus* by its dorsolateral glandular folds separated by scattered granular projections (vs. dorsal skin having faint and interrupted glandular folds).

*Nyctibatrachus pulivijayani* differs from *N. robinmoorei* by its dorsum light brown to silvery-brown with prominent dark brown spots in life (vs. dorsum greyish or orangish-brown without prominent dark spots), forearm shorter than hand length, male FAL/HAL ratio 71.4–84.8%, *N* = 5 (vs. longer, male FAL/HAL ratio 105.3–111.8%, *N* = 2), thigh nearly equal to shank, male TL/SHL ratio 94–104.5%, *N* = 5 (vs. longer, male TL/SHL ratio 127.5–130.8%, *N* = 2), and thigh nearly equal to foot, male TL/FOL ratio 95.5–106%, *N* = 5 (vs. longer, male TL/FOL ratio 122.6–130.8%, *N* = 2).

*Nyctibatrachus pulivijayani* differs from *N. sabarimalai* by its dorsal coloration light brown to silvery-brown with prominent dark brown spots in life (vs. light greyish-brown dorsum without dark brown spots), relatively larger snout-vent size, male SVL 13.3–14.9 mm, *N* = 5 (vs. male SVL 12.3–13.2 mm, *N* = 5), head width nearly equal to head length, male HW/HL ratio 98.3–102%, *N* = 5 (vs. head longer than wide, male HW/HL ratio 82.4–89.8%, *N* = 5), and relatively smaller male FAL/HAL ratio of 71.4–84.8%, *N* = 5 (vs. male FAL/HAL ratio of 85.7–96.6%, *N* = 5).

For differences with *Nyctibatrachus manalari* see comparison of that species.

**Description of holotype *(measurements in mm)*.** Adult male (SVL 13.3); head small, length subequal to its width (HW 5.1, HL 5.0); snout rounded in dorsal and lateral view, its length (SL 2.1) longer than horizontal diameter of eye (EL 1.6); loreal region acute with indistinct canthus rostralis; interorbital space flat, wider (IUE 1.9) than upper eyelid (UEW 0.7) and internarial distance (IN 1.5); nostril closer to eye (EN 0.7) than the tip of snout (NS 0.9); tympanum indistinct; vomerine ridge present, bearing small teeth, at an angle of 75° to the body axis, closer to each other than choanae, shorter than the distance between them; tongue emarginated, bearing no median lingual process. Forearm (FAL 2.3) shorter than hand length (HAL 3.0), finger length formula: I < II < IV < III, finger discs slightly wider compared to its width (FD_I_ 0.3, FW_I_ 0.2; FD_II_ 0.3, FW_II_ 0.2; FD_III_ 0.3, FW_III_ 0.2; FD_IV_ 0.3, FW_IV_ 0.2), finger disc with dorso-terminal groove, cover bifurcate distally; subarticular tubercles prominent, oval, single, all present; prepollex distinct, oval; single palmar tubercle, oval, distinct; nuptial pads present, weakly developed. Thigh length (TL 6.3) shorter than shank (SHL 6.5) and foot (FOL 6.5), relative digit lengths I < II < V < III < IV, toe discs slightly wider compared to toe width (TD_I_ 0.2, TW_I_ 0.1; TD_II_ 0.3, TW_II_ 0.2; TD_III_ 0.4, TW_III_ 0.2; TD_IV_ 0.4, TW_IV_ 0.2; TD_V_ 0.4, TW_V_ 0.2), toe discs with dorso-terminal groove, cover bifurcate distally; foot webbing absent; subarticular tubercles well developed, oval, single, all present; inner metatarsal tubercle present, oval; outer metatarsal tubercle weakly developed.

Skin of snout shagreened to sparsely granular, upper eyelids with a few prominent glandular warts especially on the margins, presence of weakly developed dorsolateral glandular folds separated by scattered granular projections; sides of the head, anterior and posterior parts of back and upper and lower parts of flank shagreened with scattered glandular projections; subocular gland indistinct; upper surface of arms and legs shagreened with weakly developed granular projections; ventral surfaces smooth; a pair of prominent femoral glands present on the ventral surface of thighs.

**Colour of holotype.** *In life*. Dorsum silvery-brown with prominent brown spots, snout lighter in colour, a dark brown stripe between the eyes demarcating a triangular cream colour patch on the snout (Fig. 5C); upper and lower parts of flank lighter in colour than the dorsum and with scattered minute black spots and larger white spots; forelimbs (including fingers) and hind limbs (including toes) light silvery-brown with dark brown transverse bands and scattered white spots (Figs. 5A and 5C). Ventral surfaces light yellowish-brown with minute black speckles, hand and foot darker in colour, throat light grey (Fig. 5F). *In preservation*. Dorsum light grey with prominent dark grey spots, lateral sides of head grey with minute black spots, upper eyelids grey (Fig. 5I); forelimbs (including fingers) and hind limbs (including toes) grey with dark grey transverse bands; anterior and posterior parts of flanks grey. Ventral surfaces light greyish-white with minute dark grey speckles, hand and foot dark grey.

**Variations.** Morphometric data from five adult males, including the holotype, is given in Table S6. Overall, the colour, markings and meristic characters of the paratypes (except ZSI/WGRC/V/A/904) are similar to the holotype. *Colour in preservation*. ZSI/WGRC/V/A/904: dorsum dark grey with less conspicuous dark grey spots, ventral surface with more prominent dark grey speckles. ZSI/WGRC/V/A/906: dorsum light grey with fewer dark grey spots, ventral surface with more prominent dark grey speckles.

**Secondary sexual characters.** *Male* (ZSI/WGRC/V/A/902), femoral glands present (Figs. 5G and 5H), nuptial pads weakly developed.

**Figure 6:**
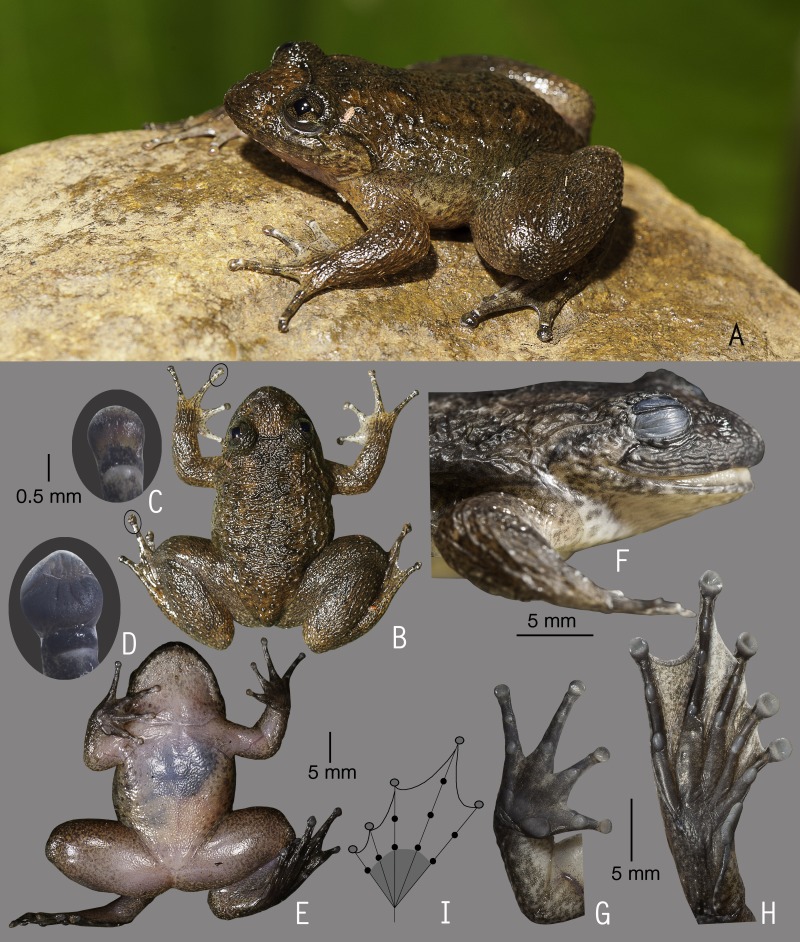
Holotype of *Nyctibatrachus radcliffei* sp. nov. (ZSI/WGRC/V/A/920). (A) Dorsolateral view, in life. (B) Dorsal view, in life. (C) Dorsal surface of third finger disc, in preservation. (D) Dorsal surface of fourth toe disc, in preservation. (E) Ventral view, in life. (F) Lateral view of head, in preservation. (G) Ventral view of hand, in preservation. (H) Ventral view of foot, in preservation. (I) Schematic illustration of foot webbing.

**Distribution and natural history.** *Nyctibatrachus pulivijayani* is currently known only from its type locality, which is located in Agasthyamala Hills, south of Palghat gap in the Western Ghats state of Kerala. Animals were found hiding under herbs and grasses on marshy ground (usually away from water) inside an evergreen forest. Males were observed calling during the day (around 11:00 h) and in the late evening (18:00 h).

**Table:** 

***Nyctibatrachus radcliffei*** sp. nov.
urn:lsid:zoobank.org:act:0F9F1491-B1E0-4F4B-9946-0E21AA7E71C8
Radcliffe’s Night Frog
(Figs. 1 and 6; Tables S1–S7)

**Holotype.** ZSI/WGRC/V/A/920, adult male, from Thiashola estate (11°13′48.2″N 76° 37′02.1″E, 1920 m), Nilgiris district, Tamil Nadu state, India, collected by SDB and SG on 09 July 2016.

**Paratypes.** ZSI/WGRC/V/A/921–922, two adult males, collected along with the holotype, and ZSI/WGRC/V/A/923–924, two adult males, collected from the same locality as holotype, by SDB and SG on 08 July 2016.

**Other material studied.** SDBDU 2014.2771, from the same locality as the type series, collected by SDB and SG on 06 October 2014, was used only for genetic identification of species.

**Etymology.** This species is named after the late Major Richard Radcliffe in recognition of his contribution towards biodiversity conservation in the Nilgiris. The species name *radcliffei* is a noun in the genitive case.

**Diagnosis.** *Nyctibatrachus radcliffei* can be distinguished from known congeners by the following combination of morphological characters: (1) medium-sized adult males (SVL 32.8–38.3 mm, *N* = 5); (2) body robust; (3) a well developed ridge extending from the lip over the tip of the snout to between the nostrils, at which point it bifurcates, producing an inverted ‘Y’; (4) wrinkled dorsal skin without prominent spinular projections; (5) third finger disc prominent (male FD_III_ 1.0–1.2, FW_III_ 0.4–0.6, *N* = 5), without dorso-terminal groove; (6) fourth toe disc prominent (male TD_IV_ 1.5–1.6, TW_IV_ 0.5–0.6, *N* = 5), with dorso-terminal groove and cover rounded distally; (7) presence of two palmar tubercles; (8) foot webbing large, fourth toe webbing extending up to the disc on either side; (9) thigh longer than shank (male TL/SHL ratio 107.1–110.2%, *N* = 5); and (10) shank nearly equal to foot length (male SHL/FOL ratio 97.6–102.9%, *N* = 5).

**Morphological comparison.** *Nyctibatrachus radcliffei* can be distinguished from its congeners *N. aliciae, N. anamallaiensis, N. athirappillyensis* sp. nov., *N. beddomii, N. deccanensis*, *N. deveni*, *N. kempholeyensis, N. manalari* sp. nov., *N. minimus, N. minor, N. periyar*, *N. pillaii*, *N. pulivijayani* sp. nov., *N. robinmoorei* sp. nov., *N. sabarimalai* sp. nov., *N. shiradi, N. vasanthi* and *N. webilla* sp. nov. by its larger adult male snout-vent size, SVL 32.8–38.3 mm (vs. smaller, male SVL 10.0–27.6 mm); differs from *N. karnatakaensis* and *N. kumbara* by its smaller adult male snout-vent size, SVL 32.8–38.3 mm (vs. larger, male SVL 42.8–63.8 mm); differs from *N. danieli*, *N. dattatreyaensis*, *N. humayuni*, *N. jog*, *N. petraeus*, *N. poocha*, *N. sanctipalustris* and *N. vrijeuni* by its fourth toe webbing extending up to the disc on either side (vs. not beyond the first subarticular tubercle on either side), third finger disc without groove, and fourth toe disc with dorso-terminal groove and cover rounded distally (vs. third finger disc with dorso-terminal groove and cover rounded distally in *N. danieli*; fourth toe disc with dorso-terminal groove and cover notched distally in *N. dattatreyaensis*; third finger disc with dorso-terminal groove and cover rounded distally in *N. humayuni, N. jog* and *N. petraeus*; third finger disc and fourth toe disc with dorso-terminal groove and cover bifurcate distally in *N. poocha*; fourth toe disc with dorso-terminal groove and cover notched distally in *N. sanctipalustris* and *N. vrijeuni*).

Because of its extensive webbing between toes, third finger and fourth toe disc morphology, and wrinkled dorsal skin, *Nyctibatrachus radcliffei* could be confused with *N. acanthodermis*, *N. gavi, N. grandis*, *N. major* and *N. sylvaticus*. *Nyctibatrachus radcliffei* may also be confused with *N. indraneili*, which occurs in the same geographical region in the Nilgiris. However, *N. radcliffei* differs from *N. acanthodermis* by its dorsal skin without prominent spinular projections (vs. prominent wrinkled skin with spinular projections), relatively smaller male snout-vent size, SVL 32.8–38.3 mm, *N* = 5 (vs. larger, male SVL 52.9–66.2 mm, *N* = 4), relatively larger male SL/HL ratio of 43.8–47.2%, *N* = 5 (vs. male SL/HL ratio of 32.8–37%, *N* = 4), relatively larger male EL/HL ratio of 26.5–32.2%, *N* = 5 (vs. male EL/HL ratio of 21.6–22.8%, *N* = 4), thigh longer than shank, male TL/SHL ratio 107.1–110.2%, *N* = 5 (vs. nearly equal, male TL/SHL ratio 99.3–100.3%, *N* = 4), shank nearly equal to foot length, male SHL/FOL ratio 97.6–102.9%, *N* = 5 (vs. longer, male SHL/FOL ratio 106.7–110.3%, *N* = 4), and fourth toe webbing extending up to the disc on either side, I1–1II1–1^+^III1–1^+^IV1–1V (vs. below, I1–1II1–1^+^III1–1^2^/_3_IV1^2^/_3_–1V).

*Nyctibatrachus radcliffei* differs from *N. gavi* by its snout rounded in lateral view (vs. nearly obtuse), relatively smaller male snout-vent size, SVL 32.8–38.3 mm, *N* = 5 (vs. larger, male SVL 49.5–57.5 mm, *N* = 2), relatively larger male SL/HL ratio of 43.8–47.2%, *N* = 5 (vs. male SL/HL ratio of 32.5–34.3%, *N* = 2), relatively smaller male FAL/HAL ratio of 61.6–69.5%, *N* = 5 (vs. male FAL/HAL ratio of 72.1–81.5%, *N* = 2), and fourth toe webbing extending up to the disc on either side, I1–1II1–1^+^III1–1^+^IV1–1V (vs. up to the first subarticular tubercle on either side: I1–1^2^/_3_II1–1^3^/_4_III1–2IV2–1V).

*Nyctibatrachus radcliffei* differs from *N. grandis* by its relatively smaller male snout-vent size, SVL 32.8–38.3 mm, *N* = 5 (vs. larger, male SVL 62.2–76.9 mm, *N* = 3), relatively smaller male HL/SVL ratio of 35.2–37.2%, *N* = 5 (vs. male HL/SVL ratio of 39.3–40.3%, *N* = 3), relatively larger male SL/HL ratio of 43.8–47.2%, *N* = 5 (vs. male SL/HL ratio of 34.5–36.3%, *N* = 3), and fourth toe webbing extending up to the disc on either side, I1–1II1–1^+^III1–1^+^IV1–1V (vs. below, I1–1II1–1^3^/_4_III1–1^3^/_4_IV1^3^/_4_–1V).

*Nyctibatrachus radcliffei* differs from *N. indraneili* by its wrinkled dorsal skin (vs. weakly wrinkled), relatively smaller male snout-vent size, SVL 32.8–38.3 mm, *N* = 5 (vs. larger, male SVL 42.5, *N* = 1), relatively larger male SL/HL ratio of 43.8–47.2%, *N* = 5 (vs. male SL/HL ratio of 33.3%, *N* = 1), eye length shorter than snout length, male EL/SL ratio 58.1–73.6%, *N* = 5 (vs. equal, male EL/SL ratio 100%, *N* = 1), fourth toe disc with dorso-terminal groove and cover rounded distally (vs. without groove), and fourth toe webbing extending up to the disc on either side, I1–1II1–1^+^III1–1^+^IV1–1V (vs. below, I1–1II1–2III1–2^−^IV2^−^–1V).

*Nyctibatrachus radcliffei* differs from *N. major* by its relatively larger male SL/HL ratio of 43.8–47.2%, *N* = 5 (vs. male SL/HL ratio of 34.3–41%, *N* = 7), relatively smaller male FAL/HAL ratio of 61.6–69.5%, *N* = 5 (vs. male FAL/HAL ratio of 74.6–86.9%, *N* = 7), thigh longer than shank, male TL/SHL ratio 107.1–110.2%, *N* = 5 (vs. nearly equal, male ratio TL/SHL 99.5–101.3%, *N* = 7), and fourth toe webbing extending up to the disc on either side, I1–1II1–1^+^III1–1^+^IV1–1V (vs. up to the first subarticular tubercle on either side, I1^1^/_3_–1^3^/_4_II1–2^1^/_3_III1–2IV2–1V).

*Nyctibatrachus radcliffei* differs from *N. sylvaticus* by its relatively larger male SL/HL ratio of 43.8–47.2%, *N* = 5 (vs. male SL/HL ratio of 35.8–37.7%, *N* = 4), relatively smaller male FAL/HAL ratio of 61.6–69.5%, *N* = 5 (vs. male FAL/HAL ratio of 76.3–79.7%, *N* = 4), and fourth toe webbing extending up to the disc on either side, I1–1II1–1^+^III1–1^+^IV1–1V (vs. up to the first subarticular tubercle on either side, I1–1^4^/_5_II1–2III1–2IV2–1V).

**Description of holotype *(measurements in mm)*.** Adult male (SVL 38.3); head wider than long (HW 14.5, HL 13.6); snout nearly rounded in dorsal view, rounded in lateral view, its length (SL 6.2) longer than horizontal diameter of eye (EL 3.6); loreal region obtuse with indistinct canthus rostralis; interorbital space flat, wider (IUE 3.8) than upper eyelid (UEW 2.3) and internarial distance (IN 2.9); nostril closer to eye (EN 2.6) than tip of snout (NS 3.3); tympanum indistinct; vomerine ridge present, bearing small teeth, at an angle of 90° to the body axis, closer to each other than choanae, longer than the distance between them; tongue moderately large, emarginated, bearing no median lingual process. Forearm (FAL 6.9) shorter than hand length (HAL 11.2), finger length formula: I < II < IV  < III, fingers with prominent discs, finger discs wide compared to finger width (FD_I_ 1.2, FW_I_ 0.6; FD_II_ 1.2, FW_II_ 0.5; FD_III_ 1.2, FW_III_ 0.4; FD_IV_ 1.0, FW_IV_ 0.4), tips rounded without groove; subarticular tubercles prominent, oval, single, all present; prepollex distinct, oval; two palmar tubercles, oval, distinct; nuptial pads present. Thigh length (TL 18.5) longer than shank (SHL 17.1) and foot (FOL 17.1), relative digit lengths I < II  < III < V < IV, toes with prominent discs, toe discs wide compared to toe width (TD_I_ 1.5, TW_I_ 0.5; TD_II_ 1.6, TW_II_ 0.6; TD_III_ 1.6, TW_III_ 0.6; TD_IV_ 1.6, TW_IV_ 0.6; TD_V_ 1.2, TW_V_ 0.5), toes discs with dorso-terminal groove, cover rounded distally; foot webbing large: I1–1II1–1^+^III1–1^+^IV1–1V, fourth toe webbing extending up to the disc on either side; subarticular tubercles well developed, oval, single, all present; inner metatarsal tubercle present, oval; outer metatarsal tubercle absent; dermal fringe along toes I and V present; a tarsal fold extending from anterior edge of the inner metatarsal tubercle.

Skin of snout shagreened to granular, upper eyelids tuberculate; sides of the head, anterior and posterior parts of back, and upper and lower parts of flank wrinkled; subocular gland prominent, extending from the posterior ventral border of the orbit towards the posterior axis of the mandibles; supratympanic fold well developed, extending from behind the eye to near the shoulder; dorsal parts of forelimb, thigh and shank with weakly developed folds or wrinkles; a well developed ridge starting from the lip and extending over the tip of the snout to between the nostrils, at which point it bifurcates, producing an inverted ‘Y’; ventral surface of throat with longitudinal folds, chest shagreened, belly shagreened to slightly wrinkled, limbs shagreened; femoral glands weakly developed.

**Colour of holotype.** *In life*. Dorsum and lateral sides of head reddish-brown with scattered blackish-brown spots, upper eyelids dark brown (Figs. 6A and 6B); forelimbs (including fingers) and hind limbs (including toes) light brown with faint brown transverse bands; anterior and posterior parts of flanks light brown. Ventral surfaces light flesh-red, sides of abdomen and hind limbs light reddish-brown with prominent black speckles; hand and foot dark grey; foot webbing light grey with minute black speckles (Fig. 6E). *In preservation*. Dorsum and lateral sides of head dark grey with scattered black spots, upper eyelids dark grey (Fig. 6F); ventral surfaces greyish-white, sides of abdomen and limbs grey with dark grey spots; hand and foot dark grey (Figs. 6G and 6H).

**Variations.** Morphometric data from five adult males, including the holotype, is given in Table S6. Overall, the colour and meristic characters of the paratypes are similar to the holotype. *Colour in preservation*. ZSI/WGRC/V/A/921, ZSI/WGRC/V/A/923–924: dorsum uniformly darker brown in colour, ventral surface with more prominent greyish-brown speckles.

**Secondary sexual characters.** *Male* (ZSI/WGRC/V/A/920), femoral glands weakly developed, nuptial pads absent.

**Distribution and natural history.** *Nyctibatrachus radcliffei* sp. nov. is currently known only from its type locality, which is located in the Nilgiris, north of Palghat gap in the southern Western Ghats state of Tamil Nadu. All the specimens were found in crevices under rocks in a hill stream inside the tea estate. In our study, we observed tadpoles of this species during the month of October 2014 and confirmed their identity using DNA. Since calls or breeding activity was not observed at the time of collection (in July), we presume that this species breeds during the early monsoon period. Collections were made between 20:00–23:00 h.

**Table:** 

**Figure 7:**
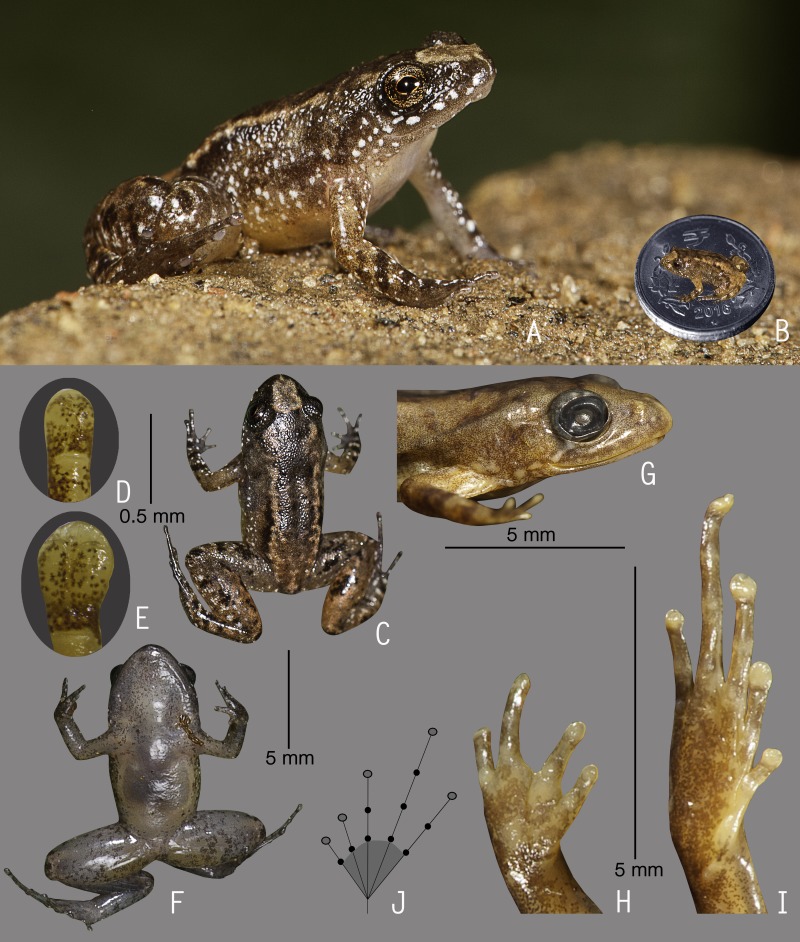
Holotype of *Nyctibatrachus robinmoorei* sp. nov. (ZSI/WGRC/V/A/925). (A) Frontolateral view, in life. (B) Size (SVL 12.2 mm) in comparison to the Indian five-rupee coin (24 mm diameter). (C) Dorsal view, in life. (D) Dorsal surface of third finger disc, in preservation. (E) Dorsal surface of fourth toe disc, in preservation. (F) Ventral view, in life. (G) Lateral view of head, in preservation. (H) Ventral view of hand, in preservation. (I) Ventral view of foot, in preservation. (J) Schematic illustration of foot webbing.

***Nyctibatrachus robinmoorei*** sp. nov.
urn:lsid:zoobank.org:act:DB413903-56B5-4D36-93B4-ABA3555CC99F
Robin Moore’s Night Frog
(Figs. 1 and 7; Tables S1–S7)

**Holotype.** ZSI/WGRC/V/A/925, adult male, from Kakkachi (08°33′02.6″N, 77°23′29.6″E, 1290 m), Tirunelveli district, Tamil Nadu state, India, collected by SDB on 30 August 2002.

**Paratype.** ZSI/WGRC/V/A/926, adult female, collected along with the holotype.

**Referred specimen.** SDBDU 2002.312, adult male, collected along with the holotype.

**Etymology.** The species is named for Dr Robin Moore, a wildlife photographer and conservationist, in appreciation of his contribution to amphibian conservation. The species name *robinmoorei* is considered as a noun in the genitive case.

**Diagnosis.** *Nyctibatrachus robinmoorei* can be distinguished from known congeners by the following combination of morphological characters: (1) miniature-sized adult males (SVL 12.2–13.4 mm, *N* = 2); (2) head width nearly equal to head length (male HW/HL ratio 97.6–100%, *N* = 2); (3) presence of weakly developed dorsolateral glandular folds separated by scattered granular projections; (4) third finger disc slightly wider than finger width (male FD_III_ 0.4–0.5, FW_III_ 0.3–0.4, *N* = 2), with dorso-terminal groove and cover bifurcate distally; (5) fourth toe disc slightly wider than toe width (male TD_IV_ 0.5–0.6, TW_IV_ 0.3, *N* = 2), with dorso-terminal groove and cover bifurcate distally; (6) presence of single palmar tubercle; (7) foot webbing absent; (8) forearm longer than hand length (male FAL/HAL ratio 105.3–111.8%, *N* = 2); (9) thigh longer than shank length (male TL/SHL ratio 127.5–130.8%, *N* = 2); and (10) thigh longer than foot length (male TL/FOL ratio 122.6–130.8%, *N* = 2).

**Morphological comparison.** *Nyctibatrachus robinmoorei* can be distinguished from all its congeners, expect *N. anamallaiensis, N. athirappillyensis* sp. nov., *N. beddomii, N. deccanensis*, *N. kempholeyensis, N. manalari* sp. nov., *N. minimus, N. minor, N. pulivijayani* sp. nov., *N. sabarimalai* sp. nov. and *N. webilla* sp. nov., by its smaller male snout-vent size, SVL 12.2–13.4 mm (vs. male SVL 18.1–76.9 mm), absence of webbing between toes (vs. present) and dorsal skin not prominently wrinkled and/or granular (vs. prominently wrinkled and/or granular); differs from *N. athirappillyensis, N. deccanensis* and *N. kempholeyensis* by absence of webbing between toes (vs. present); differs from *N. minor* by its dorsal skin without distinct dorsolateral glandular folds separated by an ‘X’ pattern on anterior half of back (vs. present); and differs from *N. webilla* by its third finger disc with dorso-terminal groove and cover bifurcate distally (vs. without dorso-terminal groove).

Because of the comparable snout-vent size, *Nyctibatrachus robinmoorei* could be confused with three previously known species *N. anamallaiensis*, *N. beddomii* and *N. minimus,* and three new species *N. manalari*, *N. pulivijayani* and *N. sabarimalai*. However, *N. robinmoorei* differs from *N. anamallaiensis* by its head nearly as wide as long, male HW/HL ratio 97.6–100%, *N* = 2 (vs. head wider than long, male HW/HL 115.7–126.1%, *N* = 5), eye length shorter than snout length, male EL/SL ratio 71.4–75%, *N* = 2 (vs. longer, male EL/SL ratio 125–140%, *N* = 5), forearm longer than hand length, male FAL/HAL ratio 105.3–111.8%, *N* = 2 (vs. shorter, male FAL/HAL ratio 72.7–83.3%, *N* = 5), and thigh longer than shank, male TL/SHL ratio 127.5–130.8%, *N* = 2 (vs. nearly equal, male TL/SHL ratio 98.4–101.6%, *N* = 5).

*Nyctibatrachus robinmoorei* differs from *N. beddomii* by its dorsolateral glandular folds separated by scattered granular projections (vs. dorsal skin finely granular), head nearly as wide as long, male HW/HL ratio 97.6–100%, *N* = 2 (vs. head wider than long, male HW/HL 106.6–129.3%, *N* = 10), and thigh longer than shank, male TL/SHL ratio 127.5–130.8%, *N* = 2 (vs. nearly equal, male TL/SHL ratio 95.9–101.6%, *N* = 10).

*Nyctibatrachus robinmoorei* differs from *N. minimus* by its dorsolateral glandular folds separated by scattered granular projections (vs. dorsal skin with faint and interrupted glandular folds), forearm longer than hand length, male FAL/HAL ratio 105.3–111.8%, *N* = 2 (vs. shorter, male FAL/HAL ratio 69–82.9%, *N* = 14), and thigh longer than shank, male TL/SHL ratio 127.5–130.8%, *N* = 2 (vs. nearly equal, male TL/SHL ratio 95.9–101.6%, *N* = 14).

*Nyctibatrachus robinmoorei* differs from *N. sabarimalai* by its head nearly as wide as long, male HW/HL ratio 97.6–100%, *N* = 2 (vs. head longer than wide, male HW/HL 82.4–89.8%, *N* = 5), forearm longer than hand length, male FAL/HAL ratio 105.3–111.8%, *N* = 2 (vs. shorter, male FAL/HAL ratio 85.7–96.6%, *N* = 5), thigh longer than shank length, male TL/SHL ratio 127.5–130.8%, *N* = 2 (vs. nearly equal, male TL/SHL ratio 98.4–101.6%, *N* = 5), and thigh longer than foot length, male TL/FOL ratio 122.6–130.8%, *N* = 2 (vs. nearly equal, male TL/FOL ratio 96.9–101.6%, *N* = 5).

For differences with *Nyctibatrachus manalari* and *N. pulivijayani* see comparison of those species.

**Description of holotype *(measurements in mm)*.** Adult male (SVL 12.2); head small, head width subequal to head length (HW 4.0, HL 4.1); snout rounded in dorsal and lateral views, its length (SL 2.0) longer than horizontal diameter of eye (EL 1.5); loreal region obtuse with indistinct canthus rostralis; interorbital space flat, wider (IUE 1.9) than upper eyelid (UEW 0.6) and internarial distance (IN 1.5); nostril closer to eye (EN 1.0) than the tip of snout (NS 1.3); tympanum indistinct; vomerine ridge present, bearing small teeth, at an angle of 85° to the body axis, closer to each other than choanae, longer than the distance between them; tongue emarginated, bearing no median lingual process. Forearm (FAL 1.9) longer than hand length (HAL 1.7), finger length formula: I < II = IV < III, finger discs slightly wide compared to finger width (FD_I_ 0.3, FW_I_ 0.2; FD_II_ 0.3, FW_II_ 0.2; FD_III_ 0.4, FW_III_ 0.3; FD_IV_ 0.3, FW_IV_ 0.2), finger disc with dorso-terminal groove, cover bifurcate distally; subarticular tubercles prominent, oval, single, all present; prepollex distinct, oval; single palmar tubercle, oval, distinct; nuptial pads present. Thigh (TL 6.5) longer than shank (SHL 5.1) and foot (FOL 5.3), relative toe lengths I < II < V <III < IV, toe discs slightly wide compared to toe width (TD_I_ 0.3, TW_I_ 0.2; TD_II_ 0.3, TW_II_ 0.2; TD_III_ 0.5, TW_III_ 0.3; TD_IV_ 0.5, TW_IV_ 0.3; TD_V_ 0.3, TW_V_ 0.2), toe discs with dorso-terminal grooves, cover bifurcate distally; foot webbing absent; subarticular tubercles well developed, oval, single, all present; inner metatarsal tubercle present, oval; outer metatarsal tubercle weakly developed.

Skin of snout shagreened to sparsely granular, upper eyelids with a few prominent glandular warts especially on the margins, presence of weakly developed dorsolateral glandular folds separated by granular projections; sides of the head, anterior and posterior parts of back, and upper and lower parts of flank shagreened with scattered glandular projections; upper surface of arms and legs with minute granular projections; ventral surfaces smooth; a pair of prominent femoral glands present on the ventral surface of thighs (Fig. 7F).

**Colour of holotype.** *In life*. Dorsum reddish-brown, with a pair of light orangish-brown longitudinal bands starting from behind the eyelids and extending up to the middle of dorsum (Fig. 7C), another light orangish-brown stripe between the eyes demarcating a triangular light orangish-brown patch on the snout; lateral sides of the head dark greyish-brown with scattered minute black speckles and prominent white spots, upper eyelids dark greyish-brown (Fig. 7C); upper and lower parts of flank light brown with scattered white spots (Fig. 7A); forelimbs (including fingers) and hind limbs (including toes) light brown with faint dark grey transverse bands. Ventral surfaces light grey with prominent minute black spots, hand and foot darker in colour (Fig. 7F). *In preservation*. Dorsum brown, with a pair of light brown longitudinal bands starting from behind the eyelids and extending up to the middle of dorsum, another light brown stripe between the eyes demarcating a triangular light brown patch on the snout; upper eyelids dark grey; forelimbs (including fingers) and hind limbs (including toes) light greyish-brown with faint grey transverse bands; anterior and posterior parts of flanks light greyish-brown. Ventral surfaces light greyish-white with minute dark grey spots.

**Variations.** Morphometric data from two adult males and an adult female, including the holotype, is given in Table S6. Overall, the colour, markings and meristic characters of the paratype and referred specimen are similar to the holotype.

**Secondary sexual characters.** *Male* (ZSI/WGRC/V/A/925), femoral glands present, nuptial pads present; *female* (ZSI/WGRC/V/A/926), small pigmented eggs present (diameter 1.7 ± 0.3mm, *N* = 15).

**Distribution and natural history.** *Nyctibatrachus robinmoorei* is currently known only from its type locality, which is located in the Kalakkad Mundanthurai Tiger Reserve, south of Palghat gap in the Western Ghats state of Tamil Nadu. Animals were collected from a marshy area covered with thick ground vegetation, close to a rivulet inside primary forest. Males were heard calling during daytime (12:00–14:00 h) and in the late evening (around 18:00 h).

**Table:** 

**Figure 8:**
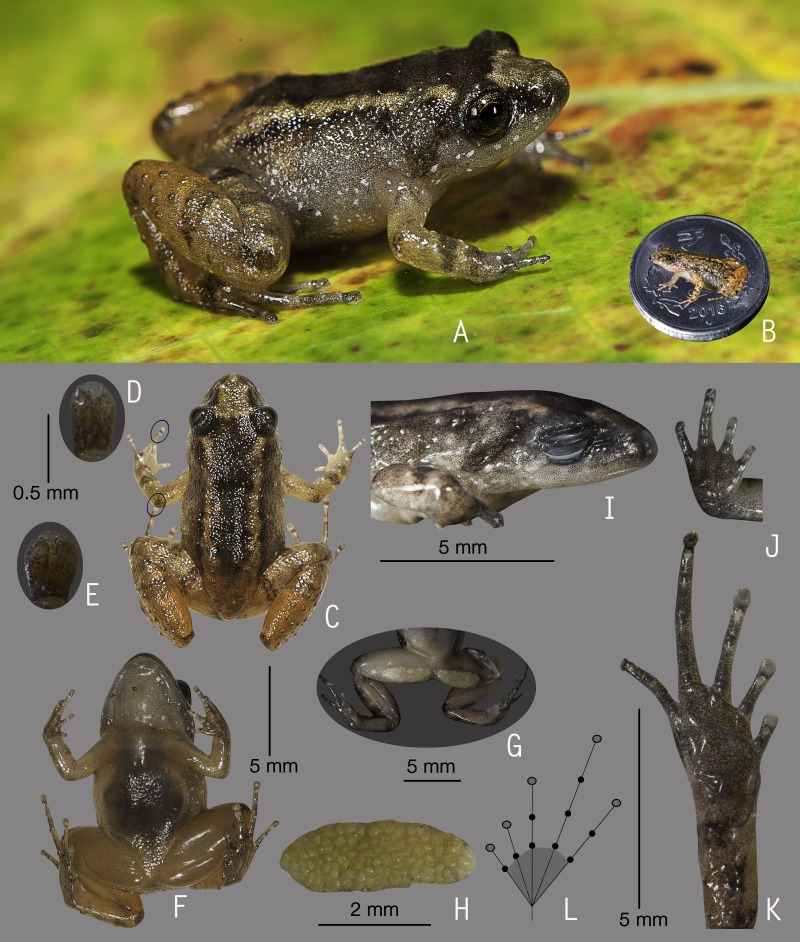
Holotype of *Nyctibatrachus sabarimalai* sp. nov. (ZSI/WGRC/V/A/927). (A) Dorsolateral view, in life. (B) Size (SVL 12.8 mm) in comparison to the Indian five-rupee coin (24 mm diameter). (C) Dorsal view, in life. (D) Dorsal surface of third finger disc, in preservation. (E) Dorsal surface of fourth toe disc, in preservation. (F) Ventral view, in life. (G) Femoral glands, in preservation. (H) Close-up of femoral glands after removal of skin showing multiple glands. (I) Lateral view of head, in preservation. (J) Ventral view of hand, in preservation. (K) Ventral view of foot, in preservation. (L) Schematic illustration of foot webbing.

**Figure 9:**
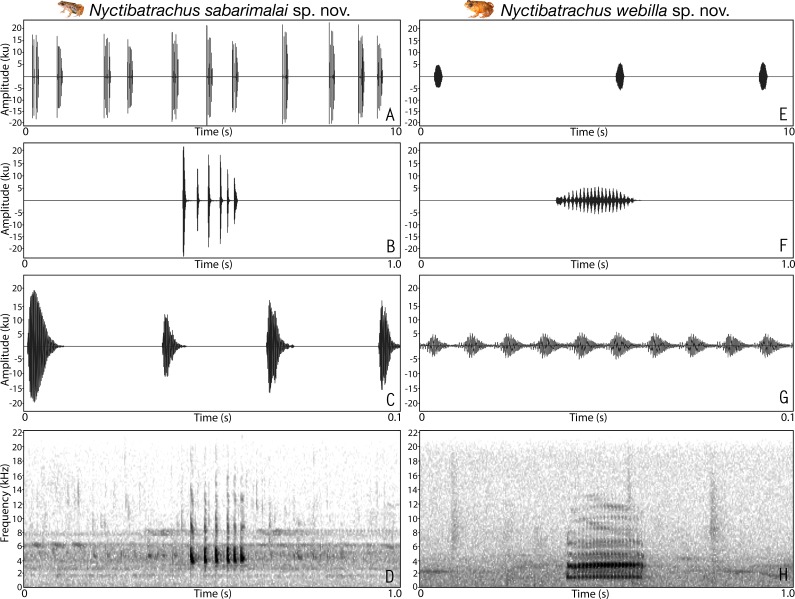
Male advertisement calls of *Nyctibatrachus sabarimalai* sp. nov. and *Nyctibatrachus webilla* sp. nov. (A–D) *Nyctibatrachus sabarimalai* sp. nov. (A) 10 s segment. (B) 1 s segment showing a single pulsatile call. (C) 0.1 s segment. (D) Spectrogram of 1 s call segment. (E–H) *Nyctibatrachus webilla* sp. nov. (E) 10 s segment. (F) 1 s segment showing a single pulsatile call. (G) 0.1 s segment. (H) Spectrogram of 1 s call segment.

***Nyctibatrachus sabarimalai*** sp. nov.
urn:lsid:zoobank.org:act:4517569C-ECCA-49C9-B1C3-A1B6E4FD6F00
Sabarimala Night Frog
(Figs. 1, 8 and 9; Tables S1–S8)

**Holotype.** ZSI/WGRC/V/A/927, adult male, from Pamba (09°24′17.6″N 77°04′11.6″E, 210 m), Pathanamthitta district, Kerala state, India, collected by SDB and SG on 17 July 2016.

**Paratypes.** ZSI/WGRC/V/A/928–931, four adult males collected along with the holotype, and ZSI/WGRC/V/A/932, adult female, collected from the same locality as holotype, by SDB, SG, RS, SS on 02 July 2015.

**Etymology.** The species is named after Sabarimala, a pilgrim site located inside the Periyar Tiger Reserve, from the surroundings of which the type series was collected. The species name is considered as a noun in the genitive case.

**Diagnosis.** *Nyctibatrachus sabarimalai* can be distinguished from known congeners by the following combination of morphological characters: (1) miniature-sized adult males (SVL 12.3–13.2 mm, *N* = 5); (2) head longer than wide (male HW/HL ratio 82.4–89.8%, *N* = 5); (3) presence of weakly developed dorsolateral glandular folds separated by scattered granular projections; (4) third finger disc slightly wider than finger width (male FD_III_ 0.3–0.4, FW_III_ 0.2–0.3, *N* = 5), with dorso-terminal groove and cover bifurcate distally; (5) fourth toe disc slightly wider than toe width (male TD_IV_ 0.5–0.6, TW_IV_ 0.3–0.4, *N* = 5), with dorso-terminal groove and cover bifurcate distally; (6) presence of single palmar tubercle; (7) foot webbing absent; (8) thigh nearly equal to shank length (male TL/SHL ratio 98.4–101.6%, *N* = 5); (9) thigh nearly equal to foot length (male TL/FOL ratio 96.9–101.6%, *N* = 5); and (10) shank nearly equal to foot length (male SHL/FOL ratio 98.4–100%, *N* = 5).

**Morphological comparison.** *Nyctibatrachus sabarimalai* can be distinguished from all its congeners, expect *N. anamallaiensis, N. athirappillyensis* sp. nov., *N. beddomii, N. deccanensis*, *N. kempholeyensis, N. manalari* sp. nov., *N. minimus, N. minor, N. pulivijayani* sp. nov., *N. robinmoorei* sp. nov. and *N. webilla* sp. nov., by its smaller male snout-vent size, SVL 12.3–13.2 mm (vs. larger, 18.1–76.9 mm), absence of webbing between toes (vs. present) and dorsal skin not prominently wrinkled and/or granular (vs. prominently wrinkled and/or granular); differs from *N. athirappillyensis, N. deccanensis* and *N. kempholeyensis* by absence of webbing between toes (vs. present); differs from *N. minor* by its dorsal skin without distinct dorsolateral glandular folds separated by an ‘X’ pattern on anterior half of back (vs. present); and differs from *N. webilla* by its third finger disc with dorso-terminal groove and cover bifurcate distally (vs. without dorso-terminal groove).

Because of the comparable snout-vent size, *Nyctibatrachus sabarimalai* could be confused with the previously known species *N. anamallaiensis*, *N. beddomii* and *N. minimus*, and the new species *N. manalari*, *N. pulivijayani* and *N. robinmoorei*. However, *N. sabarimalai* differs from *N. anamallaiensis* by its head longer than wide, male HW/HL ratio 82.4–89.8%, *N* = 5 (vs. head wider than long, male HW/HL 115.7–126.1%, *N* = 5), relatively larger male HL/SVL ratio of 38.3–41.5%, *N* = 5 (vs. male HL/SVL ratio of 30.3–37.4%, *N* = 5), relatively larger male SL/HL ratio of 35.8–40.8%, *N* = 5 (vs. male SL/HL ratio of 29.4–34.9%, *N* = 5), and eye length smaller than snout length, male EL/SL ratio 70–84.2%, *N* = 5 (vs. larger, male EL/SL ratio 125–140%, *N* = 5).

*Nyctibatrachus sabarimalai* differs from *N. beddomii* its dorsolateral glandular folds separated by scattered granular projections (vs. dorsal skin finely granular), relatively smaller snout-vent size, male SVL 12.3–13.2 mm, *N* = 5 (vs. male SVL 13.3–18.0 mm, *N* = 10), and head longer than wide, male HW/HL ratio 82.4–89.8%, *N* = 5 (vs. head wider than long, male HW/HL 106.6–129.3%, *N* = 10).

*Nyctibatrachus sabarimalai* differs from *N. minimus* its dorsolateral glandular folds separated by scattered granular projections (vs. dorsal skin with faint and interrupted glandular folds), and head longer than wide, male HW/HL ratio 82.4–89.8%, *N* = 5 (vs. head width nearly equal or larger than head length, male HW/HL 95.7–108%, *N* = 10).

For differences with *Nyctibatrachus manalari*, *N. pulivijayani* and *N. robinmoorei* see comparison of those species.

**Description of holotype *(measurements in mm)*.** Adult male (SVL 12.8); head small, longer than wide (HW 4.4, HL 4.9); snout rounded in dorsal and lateral views, its length (SL 1.9) longer than horizontal diameter of eye (EL 1.4); loreal region obtuse with indistinct canthus rostralis; interorbital space flat, wider (IUE 1.6) than upper eyelid (UEW 0.8) and internarial distance (IN 1.3); nostril closer to eye (EN 0.8) than tip of snout (NS 1.2); tympanum indistinct; vomerine ridge present, bearing numerous small teeth, at an angle of 70° to body axis, closer to each other than choanae, longer than the distance between them; tongue emarginated, bearing no median lingual process. Forearm (FAL 2.6) shorter than hand length (HAL 2.8), finger length formula: I < II < IV  < III, finger discs slightly wider compared to finger width (FD_I_ 0.2, FW_I_ 0.1; FD_II_ 0.2, FW_II_ 0.1; FD_III_ 0.3, FW_III_ 0.2; FD_IV_ 0.2, FW_IV_ 0.2), finger disc with dorso-terminal groove, cover bifurcate distally; subarticular tubercles prominent, oval, single, all present; prepollex distinct, oval; single palmar tubercle, oval, distinct; nuptial pads present. Thigh length (TL 6.7) nearly equal to shank (SHL 6.6) and foot (FOL 6.6), relative digit lengths I < II  < V II III < IV, toe discs slightly wider compared to toe width (TD_I_ 0.4, TW_I_ 0.3; TD_II_ 0.4, TW_II_ 0.3; TD_III_ 0.5, TW_III_ 0.3; TD_IV_ 0.5, TW_IV_ 0.4; TD_V_ 0.4, TW_V_ 0.3), toe disc with dorso-terminal groove, cover bifurcate distally; foot webbing absent; subarticular tubercles well developed, oval, single, all present; inner metatarsal tubercle present, oval; outer metatarsal tubercle weakly developed.

Skin of snout shagreened to granular, upper eyelids with a few prominent glandular warts especially on the margins, presence of weakly developed dorsolateral glandular folds separated by scattered granular projections; sides of head, anterior and posterior parts of back, and upper and lower parts of flank with scattered glandular projections; subocular gland indistinct; upper surface of arms and legs with minute granular projections; ventral surfaces smooth; a pair of prominent femoral glands present on the ventral surface of thighs.

**Colour of holotype.** *In life*. Dorsum dark-brown, with a pair of light brown longitudinal bands starting from behind the eyelids and extending up to the middle of dorsum, another light brown stripe between the eyes demarcating a triangular light brown patch on the snout (Fig. 8C); lateral sides of head light greyish-brown with scattered white spots, upper eyelids dark brown (Fig. 8C); upper and lower parts of flank light grey with scattered white spots; forelimbs (including fingers) and hind limbs (including toes) light brown with dark grey transverse bands. Ventral surfaces greyish-white, hand and foot lighter in color than abdomen (Fig. 8F). *In preservation*. Dorsum dark grey, with a pair of light grey longitudinal bands starting from behind the eyelids and extending up to the middle of dorsum, another light grey stripe between the eyes demarcating a triangular light grey patch on the snout; lateral sides of head light grey, anterior and posterior parts of flanks greyish-white, upper eyelids dark grey (Fig. 8I); forelimbs (including fingers) and hind limbs (including toes) light grey with grey transverse bands. Ventral surfaces greyish-white.

**Variations.** Morphometric data from five adult males and an adult female, including the holotype, is given in Table S6. Overall, the colour and meristic characters of the paratypes are similar to the holotype. *Colour in preservation*. ZSI/WGRC/V/A/930–932: dorsum lighter grey in colour with light brown markings; ZSI/WGRC/V/A/928: ventral surface of hindlimbs with a few scattered grey speckles.

**Secondary sexual characters.** *Male* (ZSI/WGRC/V/A/927), femoral glands present (Figs. 8G and 8H), nuptial pads present. *Female* (ZSI/WGRC/V/A/932), large pigmented eggs present (diameter 2.4 ± 0.5mm, *N* = 10).

**Vocalization.** Male (ZSI/WGRC/V/A/929) of *Nyctibatrachus sabarimalai* produced a single type of call. Calls were not delivered in groups and had a pulsatile temporal structure. The call had duration of 139.9 ms and six pulses were delivered at a rate of 45.6 pulses/s. The call envelope was characterized by a rise time of 1.9 ms, fall of 137.9 ms, and the overall dominant frequency of 4.4 kHz (Table S8; Figs. 9A–9D).

**Distribution and natural history.** *Nyctibatrachus sabarimalai* is currently known only from its type locality, which is located close to Sabarimala in Periyar Tiger Reserve, south of Palghat gap in the Western Ghats state of Kerala. Individuals were located under leaf litter in a shallow forest stream or under the grasses on wet rocky terrain. A calling male was found positioned next to an egg clutch (10 eggs) deposited inside a slit on a tree stump about one foot above ground. Males were observed calling both during the day (between 15:00–17:00 h) and night (20:00–22:00 h).

**Table:** 

**Figure 10:**
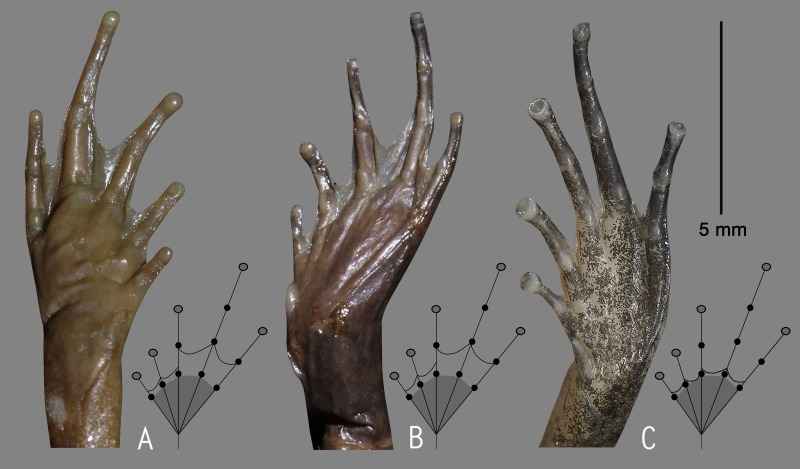
Relative degree of foot webbing in the Lectotype (NHM 1947.2.4.51) of *Nyctibatrachus deccanensis* (A), Holotype (ZSI/WGFRS/V/A 611) of *Nyctibatrachus sholai* (=*Nyctibatrachus deccanensis*) (B), and Holotype (ZSI/WGRC/V/A/933) of *Nyctibatrachus webilla* sp. nov. (C). Ventral surface of foot is shown on the left and schematic illustration on the right.

**Figure 11:**
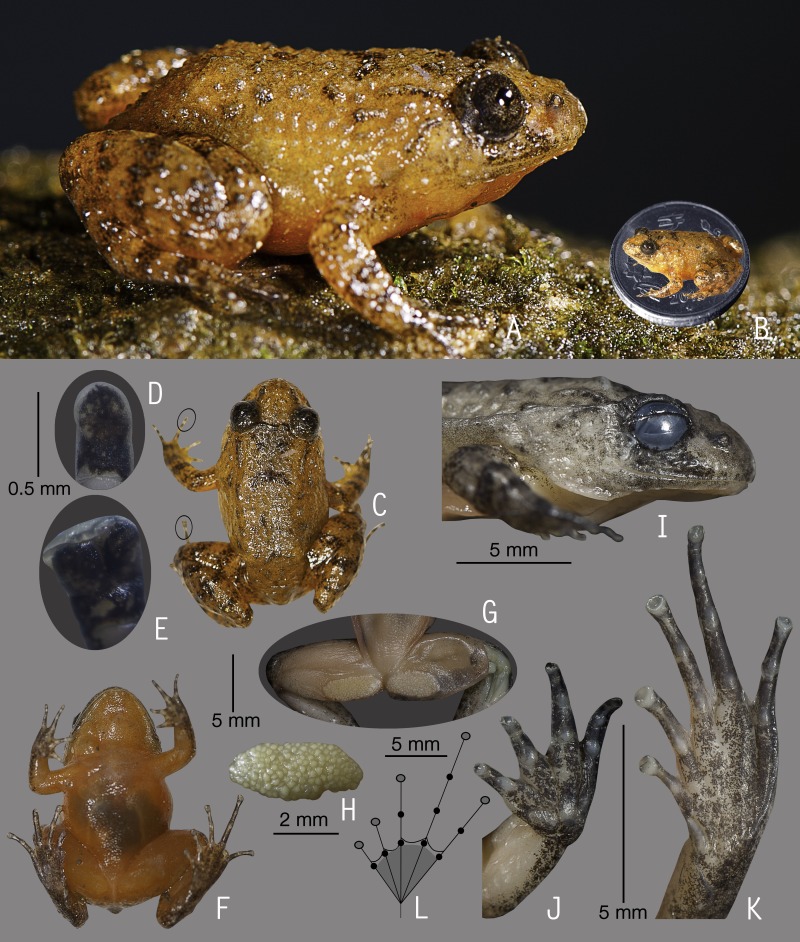
Holotype of *Nyctibatrachus webilla* sp. nov. (ZSI/WGRC/V/A/933). (A) Dorsolateral view, in life. (B) Size (SVL 20.7 mm) in comparison to the Indian five-rupee coin (24 mm diameter). (C) Dorsal view, in life. (D) Dorsal surface of third finger disc, in preservation. (E) Dorsal surface of fourth toe disc, in preservation. (F) Ventral view, in life. (G) Femoral glands, in preservation. (H) Close-up of femoral glands after removal of skin showing multiple glands. (I) Lateral view of head, in preservation. (J) Ventral view of hand, in preservation. (K) Ventral view of foot, in preservation. (L) Schematic illustration of foot webbing.

***Nyctibatrachus webilla*** sp. nov.
urn:lsid:zoobank.org:act:735BCF83-6BE0-4F2A-97E6-39BFCDA5B944
Kadalar Night Frog
(Figs. 1, 9–11; Tables S1–S8)

**Holotype.** ZSI/WGRC/V/A/933, adult male, from Kadalar (10°07′52.0″N 77°00′01.8″E, 1429 m), Idukki district, Kerala state, India, collected by SDB and SG on 08 June 2016.

**Paratypes.** ZSI/WGRC/V/A/934, adult male, collected along with the holotype, and ZSI/WGRC/V/A/935–936, two adult males, collected from the same locality as holotype, by SDB and SG on 18 August 2013.

**Etymology.** The species name is derived from the English term ‘web’ between toes and the Malayalam word ‘illa’, meaning ‘no’—referring to the prominently reduced foot webbing in this species in comparison to its close relative *Nyctibatrachus deccanensis*. The species name is treated as an invariable noun in apposition to the generic name.

**Diagnosis.** *Nyctibatrachus webilla* can be distinguished from known congeners by the following combination of morphological characters: (1) small male adult size (SVL 18.7–20.7 mm, *N* = 4); (2) head wider than long (male HW/HL ratio 110.8–117.4%, *N* = 4); (3) glandular ridges on sides of the head, anterior and posterior parts of back, upper and lower parts of flank, and a glandular ridge between the eyes; (4) third finger disc slightly wider than finger width (male FD_III_ 0.3–0.4, FW_III_ 0.2–0.3, *N* = 4), without groove; (5) fourth toe disc moderately wider than toe width (male TD_IV_ 0.6–0.7, TW_IV_ 0.3–0.4, *N* = 4), with dorso-terminal groove and cover bifurcate distally; (6) presence of two palmar tubercles; (7) foot webbing basal, fourth toe webbing just above the third subarticular tubercle on either side; (8) thigh longer than shank (male TL/SHL ratio 106.9–111.1%, *N* = 4); (9) shank shorter than foot length (male SHL/FOL ratio 90.0–93.5%, *N* = 4); and (10) bright reddish-orange ventral coloration in life.

**Morphological comparison.** *Nyctibatrachus webilla* can be distinguished from its congeners *N. acanthodermis, N. danieli, N. dattatreyaensis*, *N. gavi*, *N. grandis*, *N. humayuni*, *N. indraneili*, *N. jog*, *N. karnatakaensis*, *N. kumbara*, *N. major*, *N. periyar*, *N. petraeus*, *N. poocha*, *N. radcliffei* sp. nov., *N. sanctipalustris*, *N. sylvaticus* and *N. vrijeuni* by its smaller adult male snout-vent size, SVL 18.7–20.7 mm (vs. larger, male SVL 24.2–76.9 mm), and dorsal skin less prominently wrinkled and/or granular (vs. prominently wrinkled and/or granular); differs from *N. anamallaiensis, N. beddomii, N. manalari* sp. nov., *N. minimus, N. pulivijayani* sp. nov., *N. robinmoorei* sp. nov. and *N. sabarimalai* sp. nov. by its larger adult male snout-vent size, SVL 18.7–20.7 mm (vs. smaller, male SVL 10–18 mm); differs from *N. aliciae*, *N. deveni*, *N. pillaii*, *N. shiradi* and *N. vasanthi* by its third finger disc without groove (vs. third finger disc with dorso-terminal groove and cover notched distally in *N. aliciae*, *N. deveni* and *N. shiradi*; third finger disc with dorso-terminal groove and cover bifurcate distally in *N. pillaii* and *N. vasanthi*), and webbing between toes reduced, specifically fourth toe webbing just above the third subarticular tubercle (vs. medium webbing between toes, specifically fourth toe webbing extending well beyond the second subarticular tubercle on either side).

Because of the comparable snout-vent size, *Nyctibatrachus webilla* could be confused with previously known species *N. deccanensis*, *N. kempholeyensis* and *N. minor*, and the new species *N. athirappillyensis* sp. nov. However, *N. webilla* differs from *N. deccanensis* by its snout rounded in ventral view (vs. semi-circular), relatively larger male SL/HL ratio of 46.2–49.3%, *N* = 4 (vs. male SL/HL ratio of 30–38.7%, *N* = 15), relatively larger male EL/HL ratio of 34.4–36.2%, *N* = 4 (vs. male EL/HL ratio of 25.4–30.9%, *N* = 15), and reduced foot webbing, especially fourth toe webbing just above the third subarticular tubercle on either side, I2^−^–2^−^II2^−^–3III3^−^–4^−^IV4^−^–3^−^V (vs. up to the second subarticular tubercle on either side, I1^3^/_4_–2^−^II2^−^–3^−^III2–3IV3–2V). Since *N. webilla* is found in a region that is geographically close to the type locality of *N. sholai*, a nomen currently in the synonymy of *N. deccanensis* (Biju et al., 2011), we also compared the new species with the holotype of *N. sholai*. A comparison of relative degree of foot webbing in the holotype of *N. sholai* (=*N. deccanensis*), lectotype of *Rana pygmaea* (=*Nyctibatrachus deccanensis*) and the holotype of *N. webilla* are shown in Fig. 10.

*Nyctibatrachus webilla* differs from *N. kempholeyensis* by its third finger disc without groove (vs. with dorso-terminal groove and cover rounded distally), fourth toe disc with dorso-terminal groove and cover bifurcate distally (vs. with dorso-terminal groove and cover notched distally), reduced foot webbing, especially the fourth toe webbing just above the third subarticular tubercle on either side, I2^−^–2^−^II2^−^–3III3^−^–4^−^IV4^−^–3^−^V (vs. well beyond the second subarticular tubercle on either side, I1–2^−^II1–2^1^/_4_III1–2^1^/_2_IV2^1^/_2_–1V), and bright reddish-orange ventral coloration in life (vs. flesh or off white).

*Nyctibatrachus webilla* differs from *N. minor* by its relatively larger snout-vent size, male SVL 18.7–20.7 mm, *N* = 4 (vs. male SVL 15.4–17.9 mm, *N* = 6), third finger disc without groove (vs. with dorso-terminal groove and cover bifurcate distally), and bright reddish-orange ventral coloration in life (vs. flesh or off white).

*Nyctibatrachus webilla* differs from *N. athirappillyensis* by its third finger disc without groove (vs. with dorso-terminal groove and cover rounded distally), fourth toe disc with dorso-terminal groove and cover bifurcate distally (vs. with dorso-terminal groove and cover notched distally), reduced foot webbing, specifically the fourth toe webbing just above the third subarticular tubercle on either side, I2^−^–2^−^II2^−^–3III3^−^–4^−^IV4^−^–3^−^V (vs. well beyond the second subarticular tubercle on either side, I1–1^3^/_4_II1–2^+^III1–2^+^IV2^+^–1V), and bright reddish-orange ventral coloration in life (vs. flesh or off white). For more differences with *N. athirappillyensis*, see comparison of that species.

**Description of holotype *(measurements in mm)*.** Adult female (SVL 20.7); head small, wider than long (HW 8.1, HL 6.9); snout rounded in dorsal and lateral views, its length (SL 3.2) longer than horizontal diameter of eye (EL 2.5); loreal region acute and concave with indistinct canthus rostralis; interorbital space flat, wider (IUE 3.0) than upper eyelid (UEW 1.1) and internarial distance (IN 2.2); nostril closer to eye (EN 1.3) than the tip of snout (NS 1.9); tympanum indistinct; vomerine ridge present, bearing small teeth, at an angle of 45° to the body axis, closer to each other than choanae, longer than the distance between them; tongue moderately large, emarginated, bearing no median lingual process. Forearm (FAL 3.4) shorter than hand length (HAL 4.9), finger length formula: I < II < IV < III, finger discs slightly wider compared to finger width (FD_I_ 0.4, FW_I_ 0.3; FD_II_ 0.4, FW_II_ 0.3; FD_III_ 0.4, FW_III_ 0.3; FD_IV_ 0.4, FW_IV_ 0.3), finger discs rounded without grooves; dermal fringe present; subarticular tubercles prominent, oval, single, all present; prepollex distinct, oval; two palmar tubercles, oval, distinct; nuptial pads present. Thigh length (TL 9.9) longer than shank (SHL 9.0) and nearly equal to foot (FOL 9.8), toe discs moderately wider compared to toe width (TD_I_ 0.7, TW_I_ 0.4; TD_II_ 0.7, TW_II_ 0.4; TD_III_ 0.8, TW_III_ 0.5; TD_IV_ 0.6, TW_IV_ 0.4; TD_V_ 0.6, TW_V_ 0.4), toe disc with dorso-terminal groove, cover bifurcate distally; foot webbing basal: I2^−^–2^−^II2^−^–3III3^−^–4^−^IV4^−^–3^−^V; subarticular tubercles well developed, oval; a long and slender inner metatarsal tubercle; outer tubercle absent; tarsal fold extending from anterior edge of inner metatarsal tubercle; dermal fringe from tip of toe to heel along toe V well developed.

Skin of snout shagreened to granular, upper eyelids with a few glandular warts; sides of the head, anterior and posterior parts of back, and upper and lower parts of flank with glandular ridges; a glandular ridge between the eyes; subocular gland weakly developed, extending from the posterior ventral border of the orbit towards the posterior axis of the mandibles; supratympanic fold distinct, extending from posterior corner of upper eyelid to near the shoulder; upper surface of arms and legs with weakly developed granular projections; ventral surfaces smooth.

**Colour of holotype.** *In life.* Dorsum and lateral sides of head bright reddish-orange with scattered minute black spots, upper eyelids dark brown (Figs. 11A and 11C); forelimbs (including fingers) and hind limbs (including toes) dark reddish-brown with dark brown transverse bands; anterior and posterior parts of flanks orange. Ventral surfaces reddish-orange, hand and foot greyish-brown (Fig. 11F). *In preservation*. Dorsum and lateral sides of head light grey with scattered minute black spots and dark grey glandular folds, upper eyelids dark grey (Fig. 11I); forelimbs (including fingers) and hind limbs (including toes) grey with dark grey transverse bands; anterior and posterior parts of flanks light grey. Ventral surfaces greyish-white, margins of forelimbs and hind limbs darker grey.

**Variations.** Morphometric data from four adult males, including the holotype, is given in Table S6. Overall, the colour, markings and meristic characters of the paratypes are similar to the holotype.

**Secondary sexual characters.** *Male* (ZSI/WGRC/V/A/933), femoral glands present (Figs. 11G and 11H), nuptial pads weakly developed.

**Vocalization.** Male (ZSI/WGRC/V/A/935) of *Nyctibatrachus webilla* produced a single type of call with a pulsatile temporal structure. Calls were not delivered in groups and had uniform intervals. A typical advertisement call had a duration of 201.5 ms with 20 pulses delivered at a rate of 104 pulses/s, rise time of 52.9 ms, fall time of 92.5 ms, and the overall dominant frequency of 3.4 kHz with two broad peaks (Table S8; Figs. 9E–9H).

**Distribution and natural history.** *Nyctibatrachus webilla* is currently known only from its type locality, which is located south of Palghat gap in the Western Ghats state of Kerala. Animals were found hidden either under leaf litter or vegetation on marshy ground close to a shallow rivulet. The specific collection site was located inside a disturbed forest patch adjacent to tea estate. Males were collected and observed calling both during the day (around 10:00–12:00 h) and night (between 19:00–22:00 h).

**Figure 12:**
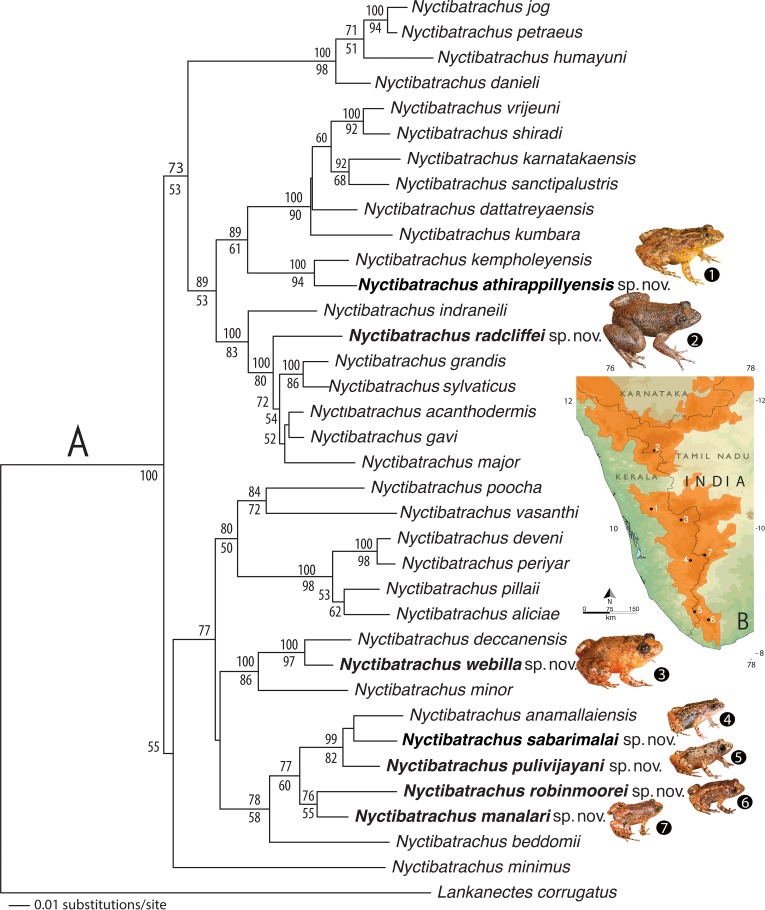
Phylogenetic relationships and distribution of the seven new *Nyctibatrachus* species described in the study. (A) Maximum Likelihood phylogram (GTR +G +I; −Ln *L* = 3621.582) for the 16S mitochondrial DNA dataset of 540 bp representing 35 *Nyctibatrachus* species (28 previously known +seven new species) from the Western Ghats of India and an outgroup taxa. Bayesian Posterior Probabilities (BPP) and RaxML bootstrap values of >50 are indicated above and below the branches, respectively. (B) Type localities of the new species in the southern Western Ghats of Peninsular India. Distribution points are referenced with species names in Fig. 12A. The Western Ghats biodiversity hotspot region is shaded orange.

### Genetic relationships

The Maximum Likelihood (ML) and Bayesian analyses (Fig. 12A) corroborate our preliminary molecular barcoding results and the detailed morphological findings. The molecular relationships recovered for known members of the genus were largely in agreement with previously published multi-gene (Van Bocxlaer et al., 2012) and mitochondrial DNA (Gururaja et al., 2014) phylogenies, allowing us to assess the genetic affinities of the new taxa with greater confidence. Relationships between all the new species and their close relatives received strong (bootstrap >  70% and BPP >  95%) to moderate (bootstrap >  50% and BPP >  70%) phylogenetic support.

Four new miniature species—*Nyctibatrachus manalari* sp. nov., *N. pulivijayani* sp. nov., *N. robinmoorei* sp. nov. and *N. sabarimalai* sp. nov.—are nested in the clade containing the two previously known *N. anamallaiensis* and *N. beddomii*, all showing closer affinities with the former. This clade corresponds with the *Nyctibatrachus beddomii* group of Van Bocxlaer et al. (2012) but is not well supported (58% BS, 78% BPP). Within this group, the clade containing *N. anamallaiensis*, *N. pulivijayani* and *N. sabarimalai* is well supported (82% BS, 99% BPP) but the relationships among these remain unresolved. *Nyctibatrachus manalari* and *N. robinmoorei* show a weakly supported sister relationship (55% BS, 76% BPP) (Fig. 12A).

The medium-sized *Nyctibatrachus athirappillyensis* sp. nov. is closely related to *N. kempholeyensis* and their relationship receives high support (94% BS, 100% BPP). On the other hand, *N. webilla* sp. nov. shows a highly supported sister relationship with *N. deccanensis* (97% BS, 100% BPP). *Nyctibatrachus minor* is sister to the clade containing *N. deccanensis* and *N. webilla*, with high support (80% BS, 100% BPP) (Fig. 12A).

The large-sized *Nyctibatrachus radcliffei* sp. nov. shows a well-supported sister relationship (80% BS, 100% BPP) with the clade clustering *N. acanthodermis*, *N. gavi*, *N. grandis*, *N. major* and *N. sylvaticus*, which corresponds with the *Nyctibatrachus major* group of Van Bocxlaer et al. (2012). *Nyctibatrachus indraneili* is sister to the clade containing *N. radcliffei* and members of the *N. major* group, with high support (80% BS, 100% BPP) (Fig. 12A).

For uncorrected pairwise distances of the new species with phylogenetically related members see Table S6.

### Call comparison

In the present study, advertisement calls of three previously known species—*Nyctibatrachus beddomii*, *N. minimus* and *N. minor*, were also recorded and herein described for the first time. Call properties are described in the Table S8, Data S1 and Fig. S2. We compared the acoustic characteristics of male advertisement calls among closely related species.

Among the four species of miniature Night Frogs (*Nyctibatrachus beddomii*, *N. manalari* sp. nov., *N. minimus* and *N. sabarimalai* sp. nov.), *N. manalari* produced only non-pulsatile calls (Figs. 3F–3I), making it distinct from the other three. The call of *N. beddomii* has two distinct parts—the non-pulsatile part 1 and the pulsatile part 2 (Figs. S2A–S2D). The pulsatile part 2 of *N. beddomii* call had nine pulses and was observed to have the fastest pulse rate of 110.0 pulses/s (Table S8). Whereas, *N. minimus* had the longest call (261.1 ms), with 18 pulses delivered at a rate of 70.8 pulses/s, and the highest overall dominant frequency of 4.9 kHz (Figs. S2E–S2H). The call of *N. sabarimalai* comprised of six pulses delivered at a rate of 45.6 pulses/s (slowest among the species being compared) and had the shortest rise time of 1.9 ms (Figs. 9A–9D).

The call patterns of *Nyctibatrachus athirappillyensis* sp. nov. and *N. webilla* sp. nov. were compared with the morphologically related *N. minor*. *Nyctibatrachus athirappillyensis* and *N. minor* had distinct call structures (Figs. 3A–3E and S2I–S2L). More specifically, the call of *N. athirappillyensis* was much longer in duration (755.7 ms) compared to *N. minor* (37.4 ms). The call of *N. athirappillyensis* also had two distinct parts—the non-pulsatile part 1 and the pulsatile part 2 (vs. only non-pulsatile calls in *N. minor*), and a lower overall dominant frequency of 3.4 kHz (vs. 4.0 kHz in *N. minor*) (Figs. 3A–3E and S2I–S2L; Table S8). On the other hand, *N. webilla* produced a single type of pulsatile call (vs. only non-pulsatile calls in *N. minor*) (Figs. 9E–9H and S2I–S2L). The call duration, call rise time and call fall time for *N. webilla* were relatively longer, 201.5 ms, 52.9 ms and 92.5 ms, respectively (vs. much shorter in *N. minor*, 37.4 ms, 1.3 ms, 35.9 ms, respectively), while the overall dominant frequency was lower, 3.4 kHz (vs. higher in *N. minor*, 4.0 kHz). The call of *N. webilla* differed from that of *N. athirappillyensis* as it produced a single type of pulsatile call (vs. call with two distinct parts—the non-pulsatile part 1 and the pulsatile part 2 in *N. athirappillyensis*) (Figs. 3A–3E and Figs. 9E–9H). *Nyctibatrachus webilla* delivered 20 pulses at a much faster rate of 104 pulses/s (vs. a higher number of pulses delivered at a slower rate, 25 pulses at a rate of 42.2 pulses/s in the pulsatile part 2 of *N. athirappillyensis*). The call duration of 201.5 ms and call fall time of 92.5 ms were much shorter in *N. webilla* (vs. relatively longer in *N. athirappillyensis*, 595.8 ms and 593.5 ms, respectively), whereas the call rise time was relatively longer (vs. shorter in *N. athirappillyensis*, 1.7 ms) (Table S8). Furthermore, the vocal repertoires of *N. athirappillyensis, N. minor* and *N. webilla* were distinct from that of *N. kempholeyensis*, as these three species produced only single type of calls (vs. two type of calls, Type I and Type II calls, in *N. kempholeyensis*) (Gururaja et al., 2014). Other call parameters could not be compared with *N. kempholeyensis* since the methodology and terminologies used for call analysis by Gururaja et al. (2014) differ from the present study.

## Discussion

After this study, the number of recognised *Nyctibatrachus* species has increased from 28 to 35. This surge clearly indicates that several more species remain to be discovered and formally described even in recently well-studied anuran groups of the Western Ghats. Four out of seven new species in our study are among the smallest known Indian frogs (male SVL 12.2–15.4 mm). Until now, only three miniature species were known in the genus (*N. anamallaienesis*, *N. beddomii* and *N. minimus*; male SVL 10.0–18.0; Biju et al., 2011). Interestingly, while erecting the genus *Nyctibatrachus*, Boulenger (1882) had also proposed a separate genus *Nannobatrachus* to accommodate the small-sized *Nannobatrachus beddomii* (=*Nyctibatrachus beddomii*), which was later synonymized under the former due to lack of morphological (Dubois, 1987 “1986”; Shaffer, 1988) and phylogenetic (Van Bocxlaer et al., 2012) support for the grouping. While future integrated studies may provide better insights regarding the taxonomic placement of this group, our discovery of several more miniature Night Frog species suggests that the diversity of diminutive forms is much higher than currently known. These frogs are usually easy to overlook not only because of the small size but also due to their secretive terrestrial habitats (usually under wet soil and dense ground vegetation) and insect-like calls. We also found these species to be fairly common and abundant at their respective collection localities. Hence, we believe that dedicated surveys can yield more species of miniaturized frogs from the Western Ghats in the future. Further studies will also be required to gain a better understanding about the evolutionary advantages of miniaturization and adaptation to terrestrial life within family Nyctibatrachidae, which is largely comprised of robust torrential frogs.

Despite recent discoveries, only a few studies have explored the vocal repertoire of *Nyctibatrachus* frogs (Kuramoto & Joshy, 2001; Gururaja et al., 2014; Willaert et al., 2016). Study of advertisement calls can provide useful insights on various aspects such as reproductive behaviour, evolutionary relationships and speciation (e.g., Gerhardt & Huber, 2002; Ryan, 2001). Although, our study does not carry out descriptive statistics and correlation of calls with other parameters (e.g., body size and temperature) due to availability of few call recordings per species, the typical call characteristics presented for seven *Nyctibatrachus* species will still be a useful resource for any future acoustic or integrative studies on this group.

The population status of the newly described species is also likely to be of concern, especially in the case of *Nyctibatrachus athirappillyensis* sp. nov., *N. radcliffei* sp. nov., *N. sabarimalai* sp. nov. and *N. webilla* sp. nov., which were collected outside National parks and sanctuaries (except *N. sabarimalai*). *Nyctibatrachus radcliffei* and *N. webilla* were found inside private or state-owned plantation areas facing threats such as habitat disturbance, modification and fragmentation. The type locality of *N. athirappillyensis*, is in close vicinity of the Athirappilly waterfall, which despite being inside a reserved forest is disturbed by anthropogenic activities. On the other hand, *N. sabarimalai*, which is found inside the protected Periyar tiger reserve, could also be facing similar threats since the type series was collected from an area close to the Sabarimala pilgrimage centre that attracts considerable anthropogenic disturbance. Besides this, all the new species are currently known only from their type localities, which are restricted to the southern Western Ghats. Six species (*N. athirappillyensis*, *N. manalari*, *N. pulivijayani*, *N. robinmoorei*, *N. sabarimalai* and *N. webilla*) are probably endemic to regions south of the Palghat gap (Fig. 12B), at elevations ranging from 530–1,555 m asl. Whereas, *N. radcliffei* is found in high elevation (above 1,900 m asl) mountain streams in the Nilgiris located north of the Palghat gap (Fig. 12B). Populations of many morphologically related species sampled in our study (such as *N. anamallaiensis*, *N. manalari* and *N. sabarimalai*; *N. beddomii*, *N. pulivijayani* and *N. robinmoorei*; or *N. indraneili* and *N. radcliffei*) were found in close vicinities, suggesting the role of factors other than geographical distance in diversification of *Nyctibatrachus* frogs. Further surveys are essential since little is known about the geographical limits, natural history, population size and habitat requirements of all the new species. This information will also be required to assess the threats and conservation needs of each species. Until a better understanding is gathered for evaluating the IUCN conservation status of the seven new taxa, we propose these to be considered as Data Deficient (DD).

In the age of rapid discoveries of amphibian species from the Western Ghats, especially in endemic and ancient lineages (e.g., Biju et al., 2011; Biju et al., 2014a), the task of protecting and conserving these evolutionarily significant relic frogs is also greater. The Western Ghats is a globally recognised biodiversity hotspot and there is growing evidence that this region is indeed a global amphibian hotspot. Therefore, studies that identify and document species diversity will strengthen the understanding required for conservation prioritization of threatened amphibians of the Western Ghats.

##  Supplemental Information

10.7717/peerj.3007/supp-1 Figure S1 Neighbor-Joining (NJ) tree based on Kimura-2-parameter model for 16S mitochondrial gene sequences, representing all the 28 previously known *Nyctibatrachus* species, seven newly sampled populations from the Western Ghats and an outgroup taxa

10.7717/peerj.3007/supp-2 Figure S2 Male advertisement calls of *Nyctibatrachus beddomii*, *N. minimus* and *N. minor*

10.7717/peerj.3007/supp-3 Table S1 List of DNA sequences used in the study

10.7717/peerj.3007/supp-4 Table S2 Uncorrected pairwise distances between 16S mitochondrial gene sequences of the new and phylogenetically related *Nyctibatrachus* species

10.7717/peerj.3007/supp-5 Table S3 Factor loadings, eigenvalues and percent variance from Principal component analysis based on nine size-corrected morphometric variables

10.7717/peerj.3007/supp-6 Table S4 Scores for Discriminant function analysis of principal components resulting from nine size-corrected morphometric variables of adult male specimens

10.7717/peerj.3007/supp-7 Table S5 Classification matrices from the discriminant function analyses

10.7717/peerj.3007/supp-8 Table S6 Morphometric measurements (in mm) of the adult specimens of seven new *Nyctibatrachus* species described in the study

10.7717/peerj.3007/supp-9 Table S7 Diagnostic characters for the new and morphologically related *Nyctibatrachus* species

10.7717/peerj.3007/supp-10 Table S8 Call properties of seven *Nyctibatrachus* species measured from single calls

10.7717/peerj.3007/supp-11 Data S1 Call descriptions for three previously known *Nyctibatrachus* species

## Additional Information and Declarations

### Field Study Permissions

The following information was supplied relating to field study approvals (i.e., approving body and any reference numbers): The study was conducted with permissions and following guidelines from the responsible authorities in the State Forest Departments of Kerala and Tamil Nadu (Study permits: No.WL12-1830/2009, No.WL10-2606/12, No.WL10-25421/2014, No.67254/2001/WL5). Research received ethical approval from Department of Environmental Studies, University of Delhi (DES/1020 dated 9 February 2015), India.

### Data Availability

The following information was supplied regarding data availability: DNA sequences are deposited in the NCBI Genbank (https://www.ncbi.nlm.nih.gov/genbank/) under accession numbers KY447300 –KY447307.

### New Species Registration

The following information was supplied regarding the registration of a newly described species: Publication LSID: urn:lsid:zoobank.org:pub:B27CCEEE-DA57-4CE6-B91F-7F799D3A8B9A *Nyctibatrachus athirappillyensis*; urn:lsid:zoobank.org:act:4756A24F-60E1-4B85-956F-5FC3CE6B876F *Nyctibatrachus manalari*; urn:lsid:zoobank.org:act:9B10F3DA-07E4-4A78-B96B-C76C3C1578F6 *Nyctibatrachus pulivijayani*; urn:lsid:zoobank.org:act:E4F167C1-8340-4779-8CEF-622AC7AB350C *Nyctibatrachus radcliffei*; urn:lsid:zoobank.org:act:0F9F1491-B1E0-4F4B-9946-0E21AA7E71C8 *Nyctibatrachus robinmoorei*; urn:lsid:zoobank.org:act:DB413903-56B5-4D36-93B4-ABA3555CC99F *Nyctibatrachus sabarimalai*; urn:lsid:zoobank.org:act:4517569C-ECCA-49C9-B1C3-A1B6E4FD6F00 *Nyctibatrachus webilla*; urn:lsid:zoobank.org:act:735BCF83-6BE0-4F2A-97E6-39BFCDA5B944
