# Supplementary material for: Seven new species of Night Frogs (Anura, Nyctibatrachidae) from the Western Ghats Biodiversity Hotspot of India, with remarkably high diversity of diminutive forms
Source: PeerJ. 2017 Feb 21;5:e3007. doi: 10.7717/peerj.3007 (PMC5322763; doi:10.7717/peerj.3007)
Supplement: Figure S1 [file peerj-05-3007-s001.pdf]

Supplemental information: **Figures**

## Seven new species of Night Frogs (Anura, Nyctibatrachidae) from the Western Ghats Biodiversity Hotspot of India, with remarkably high diversity of diminutive forms

Sonali Garg, Robin Suyesh, Sandeep Sukesan and S D Biju

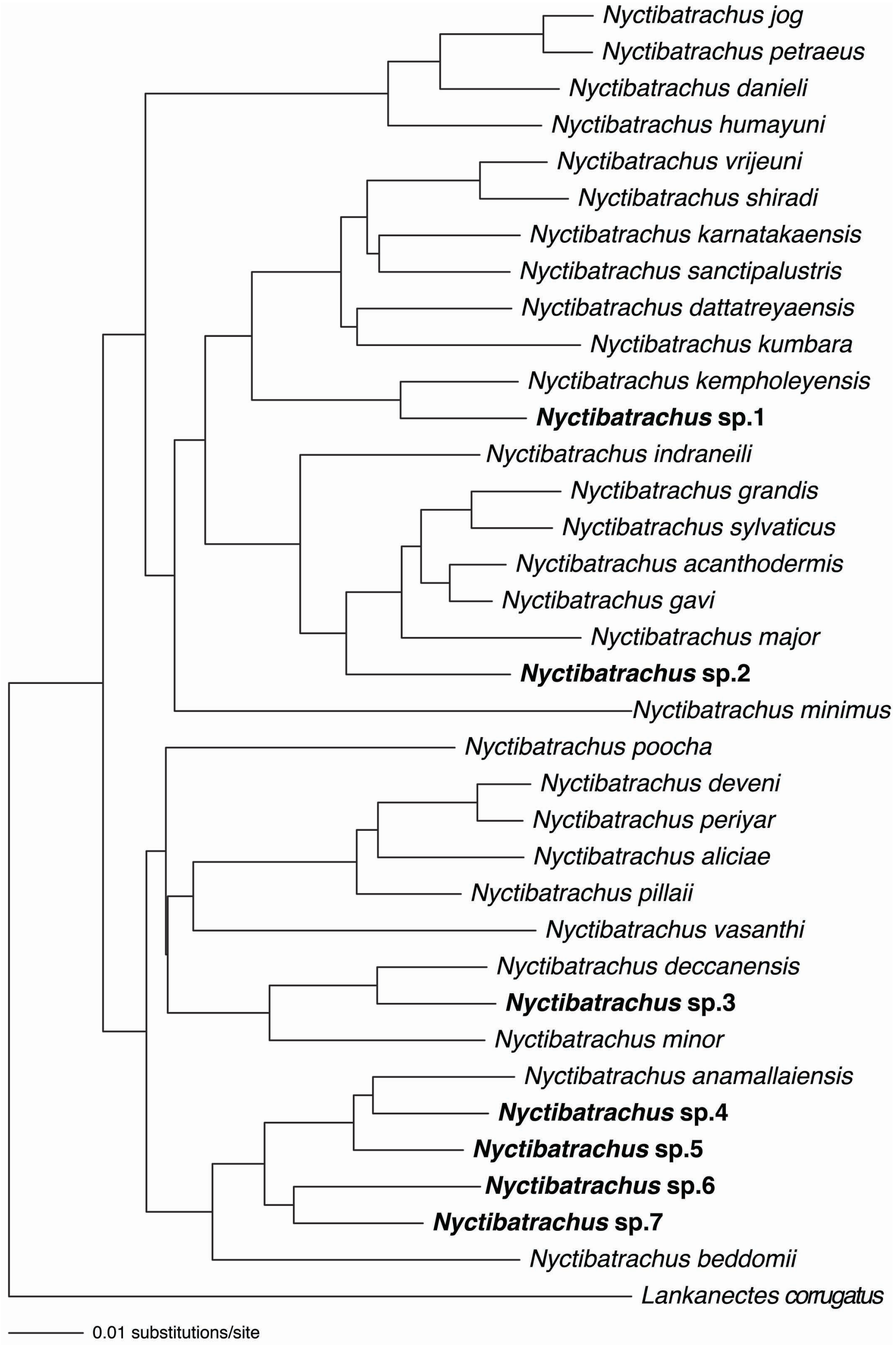


**Figure S1 Neighbor-Joining (NJ) tree based on Kimura-2-parameter model for 16S mitochondrial gene sequences, representing all the 28 previously known *Nyctibatrachus* species, seven newly sampled populations from the Western Ghats and an outgroup taxa.**
