# Supplementary material for: Seven new species of Night Frogs (Anura, Nyctibatrachidae) from the Western Ghats Biodiversity Hotspot of India, with remarkably high diversity of diminutive forms
Source: PeerJ. 2017 Feb 21;5:e3007. doi: 10.7717/peerj.3007 (PMC5322763; doi:10.7717/peerj.3007)
Supplement: Figure S2 [file peerj-05-3007-s002.pdf]

Supplemental information: **Figure**

Seven new species of Night Frogs (Anura, Nyctibatrachidae) from the Western Ghats Biodiversity Hotspot of India, with remarkably high diversity of diminutive forms

Sonali Garg, Robin Suyesh, Sandeep Sukesan and S D Biju

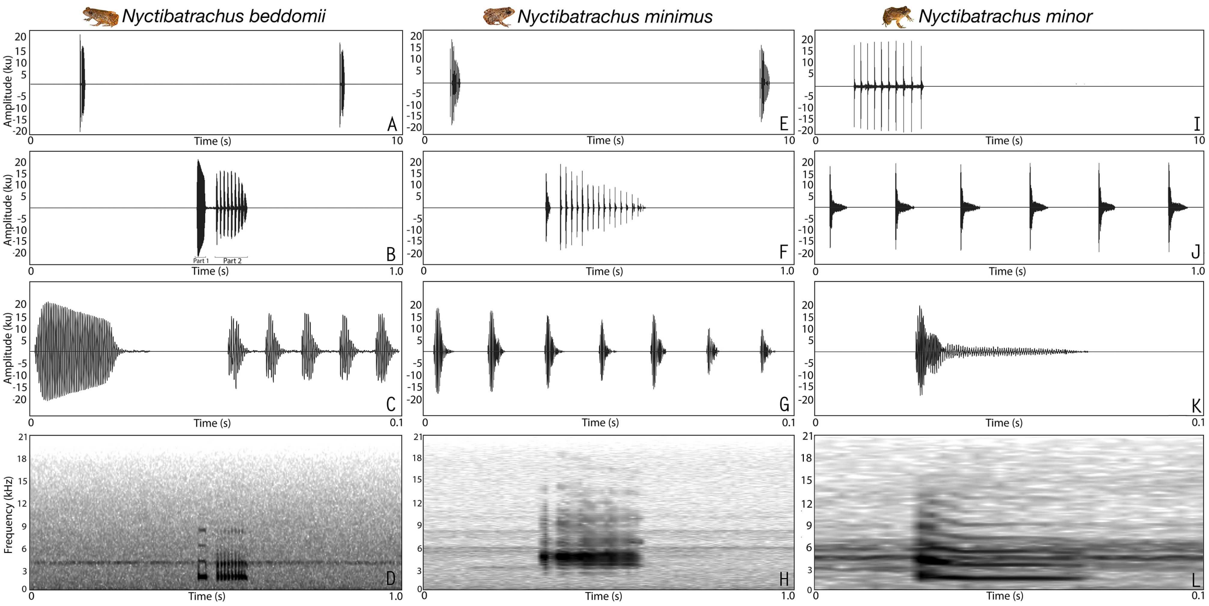

Figure S2 Male advertisement calls of *Nyctibatrachus beddomii*, *N. minimus* and *N. minor*. (A–D) *Nyctibatrachus beddomii*. (A) 10 s segment. (B) 1 s segment showing parts 1 and 2 of a single call. (C) 0.1 s segment showing the non-pulsatile part 1 and pulsatile part 2 of a single call. (D) Spectrogram of 1 s call segment. (E–H) *Nyctibatrachus minimus*. (E) 10 s segment. (F) 1 s segment showing a single pulsatile call. (G) 0.1 s segment. (H) Spectrogram of 1 s call segment. (I–L) *Nyctibatrachus minor*. (I) 10 s segment. (J) 1 s segment. (K) 0.1 s segment showing a single non-pulsatile call. (L) Spectrogram of 0.1 second segment.
