# Supplementary material for: Seven new species of Night Frogs (Anura, Nyctibatrachidae) from the Western Ghats Biodiversity Hotspot of India, with remarkably high diversity of diminutive forms
Source: PeerJ. 2017 Feb 21;5:e3007. doi: 10.7717/peerj.3007 (PMC5322763; doi:10.7717/peerj.3007)
Supplement: Table S1 [file peerj-05-3007-s003.pdf]

Supplemental information: **Tables**

Seven new species of Night Frogs (Anura, Nyctibatrachidae) from the Western Ghats Biodiversity Hotspot of India, with remarkably high diversity of diminutive forms

Sonali Garg, Robin Suyesh, Sandeep Sukesan and S D Biju

Table S1. List of DNA sequences used in the study.

Species	Collection locality	Voucher number	Accession number	Reference
N. acanthodermis	Nelliyampathi, Kerala	SDB 23	JN644915	Van Bocxlaer *et al*. 2012
N. aliciae	Ponmudi, Kerala	SDB 1142	JN644893	Van Bocxlaer *et al*. 2012
N. anamallaiensis	Valparai, Tamil Nadu	SDB 40321	JN644899	Van Bocxlaer *et al*. 2012
N. athirappillyensis	Thavalakuzhipara, Vazhachal, Kerala	ZSI/WGRC/V/A/891	KY447300	Present study
N. beddomii	Ponmudi, Kerala	SDB (DEL) 1479	JN644913	Van Bocxlaer *et al*. 2012
N. danieli	Koyna, Maharashtra	SDB (Varad) E2	JN644902	Van Bocxlaer *et al*. 2012
N. dattatreyaensis	Chikmalagur-Kemmangudi road, Karnataka	SDB (DEL) 0267	JN644891	Van Bocxlaer *et al*. 2012
N. deccanensis	Eravikulam, Kerala	SDB 1056	JN644911	Van Bocxlaer *et al*. 2012
N. deveni	Nelliyampathi, Kerala	SDB 25	JN644895	Van Bocxlaer *et al*. 2012
N. gavi	Gavi, Kerala	SDB 4836	JN644918	Van Bocxlaer *et al*. 2012
N. grandis	Thirunelli, Kerala	SDB 40360	JN644904	Van Bocxlaer *et al*. 2012
N. humayuni	Koyna, Maharashtra	SDB (Varad) 8	JN644901	Van Bocxlaer *et al*. 2012
N. indraneili	Longwood shola, Tamil Nadu	SDB 4017	JN644909	Van Bocxlaer *et al*. 2012
N. jog	Jog Falls, Karnataka	SDB 40144	JN644900	Van Bocxlaer *et al*. 2012
N. karnatakaensis	Kudremukh, Karnataka	SDB 1098	JN644910	Van Bocxlaer *et al*. 2012
N. kempholeyensis	Kempholay, Karnataka	SDBDU 2012.53	KY447307	Present study
N. kumbara	–	Isolate A1	KF935242	Gururaja *et al*. 2014
N. major	India	–	AF249052	Bossuyt & Milinkovitch 2000
N. manalari	Upper Manalar, Periyar TR, Kerala	SDBDU 2015.2994	KY447301	Present study
N. minimus	Kalpetta, Kerala	SDB 33	JN644896	Van Bocxlaer *et al*. 2012
N. minor	Ponmudi, Kerala	SDB 1146	JN644908	Van Bocxlaer *et al*. 2012
N. periyar	Vallakadavu check post, Thekkady, Kerala	SDB 4813	JN644897	Van Bocxlaer *et al*. 2012
N. petraeus	Amboli, Maharashtra	SDB 2004-023	JN644912	Van Bocxlaer *et al*. 2012
N. pillaii	Sengalthery, Tamil Nadu	SDB 40286	JN644892	Van Bocxlaer *et al*. 2012
N. poocha	Valparai, Tamil Nadu	SDB 1175	JN644907	Van Bocxlaer *et al*. 2012
N. pulivijayani	Pandipath, Agasthyamala, Kerala	ZSI/WGRC/V/A/906	KY447302	Present study

N. radcliffei	Thiashola estate, Nilgiris, Tamil Nadu	SDBDU 2014.2771	KY447303	Present study
N. robinmoorei	Kakkachi, Tamil Nadu	ZSI/WGRC/V/A/925	KY447304	Present study
N. sabarimalai	Pamba, Kerala	ZSI/WGRC/V/A/932	KY447305	Present study
N. sanctipalustris	Thalakkaveri, Karnataka	SDB 6012	JN644917	Van Bocxlaer *et al*. 2012
N. shiradi	Sakleshpur-Gundia road, Karnataka	SDB (DEL) 0233	JN644898	Van Bocxlaer *et al*. 2012
N. sylvaticus	Sakleshpur, Karnataka	SDB 4672	JN644916	Van Bocxlaer *et al*. 2012
N. vasanthi	Kakkachi, Tamil Nadu	SDB 2044	JN644894	Van Bocxlaer *et al*. 2012
N. vrijeuni	Wayanad, Kerala	SDB 3028	JN644905	Van Bocxlaer *et al*. 2012
N. webilla	Kadalar, Kerala	ZSI/WGRC/V/A/935	KY447306	Present study
Lankanectes corrugatus	Sri Lanka	VUB 0106	AY948730	Roelants *et al*. 2007
