# Supplementary material for: Seven new species of Night Frogs (Anura, Nyctibatrachidae) from the Western Ghats Biodiversity Hotspot of India, with remarkably high diversity of diminutive forms
Source: PeerJ. 2017 Feb 21;5:e3007. doi: 10.7717/peerj.3007 (PMC5322763; doi:10.7717/peerj.3007)
Supplement: Table S2 [file peerj-05-3007-s004.pdf]

Supplemental information: **Tables**

Seven new species of Night Frogs (Anura, Nyctibatrachidae) from the Western Ghats Biodiversity Hotspot of India, with remarkably high diversity of diminutive forms

Sonali Garg, Robin Suyesh, Sandeep Sukesan and S D Biju

Table S2. Uncorrected pairwise distances between 16S mitochondrial gene sequences of the new and phylogenetically related *Nyctibatrachus* species.

Species		Genetic distance
N. athirappillyensis	*N. kempholeyensis*	3.2 %
N. manalari	*N. anamallaiensis*	4.1 %
N. manalari	*N. beddomii*	6.4 %
N. manalari	*N. pulivijayani*	4.7 %
N. manalari	*N. robinmoorei*	4.1 %
N. manalari	*N. sabarimalai*	5.1 %
N. pulivijayani	*N. anamallaiensis*	3.4 %
N. pulivijayani	*N. beddomii*	7.4 %
N. pulivijayani	*N. robinmoorei*	6.0 %
N. pulivijayani	*N. sabarimalai*	3.4 %
N. robinmoorei	*N. anamallaiensis*	6.6 %
N. robinmoorei	*N. beddomii*	7.2 %
N. robinmoorei	*N. sabarimalai*	5.5 %
N. sabarimalai	*N. anamallaiensis*	3.4 %
N. sabarimalai	*N. beddomii*	7.2 %
N. radcliffei	*N. acanthodermis*	4.1 %
N. radcliffei	*N. gavi*	3.9 %
N. radcliffei	*N. grandis*	4.9 %
N. radcliffei	*N. indraneili*	6.4 %
N. radcliffei	*N. major*	5.6 %
N. radcliffei	*N. sylvaticus*	4.5 %
N. webilla	*N. deccanensis*	3.0 %
N. webilla	*N. minor*	5.3 %
