# Supplementary material for: Seven new species of Night Frogs (Anura, Nyctibatrachidae) from the Western Ghats Biodiversity Hotspot of India, with remarkably high diversity of diminutive forms
Source: PeerJ. 2017 Feb 21;5:e3007. doi: 10.7717/peerj.3007 (PMC5322763; doi:10.7717/peerj.3007)
Supplement: Table S3 [file peerj-05-3007-s005.pdf]

Supplemental information: **Tables**

Seven new species of Night Frogs (Anura, Nyctibatrachidae) from the Western Ghats Biodiversity Hotspot of India, with remarkably high diversity of diminutive forms

Sonali Garg, Robin Suyesh, Sandeep Sukesan and S D Biju

Table S3. Factor loadings, eigenvalues and percent variance from Principal component analysis based on nine size-corrected morphometric variables. Values in bold indicate variables with the highest loadings for principal components of eigenvalue >1.0.

A. Principal component analysis for adult male specimens of *Nyctibatrachus anamallaiensis*, *N. beddomii*, *N. manalari* sp. nov., *N. minimus*, *N. pulivijayani* sp. nov., *N. robinmoorei* sp. nov. and *N. sabarimalai* sp. nov.

Variable	PC 1	PC 2	PC 3	PC 4	PC 5	PC 6	PC 7	PC 8	PC 9
HW/HL	**-0.707684**	0.537753	-0.007675	0.177942	-0.039805	-0.194227	-0.320702	-0.139406	0.129190
SL/HL	-0.319729	0.120961	**-0.750394**	-0.364694	-0.362169	-0.144721	0.179465	-0.044966	0.026635
EL/HL	-0.577676	0.529307	0.152759	-0.144676	0.487535	-0.008831	0.306203	-0.100968	-0.011596
HL/SVL	**0.883365**	-0.286702	0.081370	0.130864	-0.024723	-0.045818	0.142086	-0.247624	0.171772
FAL/SVL	0.395666	0.073323	**-0.713482**	0.183194	0.452784	0.271865	-0.120385	0.024394	0.038000
HAL/SVL	0.472202	**0.663420**	-0.002604	0.455631	-0.113895	-0.203441	0.165228	0.200987	0.085062
TL/SVL	0.555910	0.173067	0.142470	**-0.734451**	0.180370	-0.162599	-0.122880	0.112010	0.121154
SHL/SVL	**0.805489**	0.428291	-0.085784	-0.024231	0.024030	-0.289056	-0.094541	-0.128841	-0.223918
FOL/SVL	0.289210	**0.673527**	0.153659	-0.171779	-0.295575	0.564882	-0.014282	-0.054159	-0.002366
Eigenvalue	3.138104	1.778701	1.153437	1.013395	0.709513	0.605229	0.315041	0.166038	0.120542
% Total variance	34.86782	19.76335	12.81597	11.25994	7.88348	6.72476	3.50045	1.84487	1.33936
Cum Eigenvalue	3.138104	4.916805	6.070242	7.083637	7.793150	8.398379	8.713419	8.879458	9.000000
Cumulative %	34.8678	54.6312	67.4471	78.7071	86.5906	93.3153	96.8158	98.6606	100.0000

B. Principal component analysis for adult male specimens of *Nyctibatrachus athirappillyensis* sp. nov., *N. deccanensis*, *N. kempholeyensis*, *N. minor* and *N. webilla* sp. nov.

Variable	PC 1	PC 2	PC 3	PC 4	PC 5	PC 6	PC 7	PC 8	PC 9
HW/HL	**0.660752**	0.166926	0.388913	-0.428236	-0.416979	-0.033946	0.018804	0.065872	-0.145559
SL/HL	-0.064299	**0.900245**	-0.055203	0.048573	0.295055	0.041641	0.281433	-0.030294	-0.105374
EL/HL	0.428554	**0.796440**	-0.033707	-0.078817	0.138277	-0.105144	-0.377770	-0.002179	0.042263
HL/SVL	**-0.876191**	-0.303083	-0.100261	-0.064639	0.187146	-0.054974	-0.196447	-0.026624	-0.221028
FAL/SVL	-0.391120	-0.111422	**0.754887**	-0.355426	0.362979	0.026719	0.008018	-0.031211	0.070157
HAL/SVL	-0.348801	0.228576	0.487811	**0.715754**	-0.183939	0.178351	-0.086366	0.041442	-0.031746
TL/SVL	**-0.716760**	0.291517	-0.164680	-0.354776	-0.264338	0.401460	-0.052786	-0.113768	0.038896
SHL/SVL	**-0.901777**	0.231697	-0.104569	-0.164330	-0.048389	-0.063764	0.025723	0.292601	0.049917
FOL/SVL	**-0.797749**	0.257290	0.101360	0.035869	-0.305754	-0.407282	0.057895	-0.146283	0.041507
Eigenvalue	3.630085	1.834006	1.021631	0.988924	0.646362	0.380602	0.275183	0.128619	0.094588
% Total variance	40.33427	20.37784	11.35146	10.98805	7.18180	4.22891	3.05759	1.42910	1.05098
Cum Eigenvalue	3.630085	5.464091	6.485722	7.474646	8.121008	8.501610	8.776793	8.905412	9.000000
Cumulative %	40.3343	60.7121	72.0636	83.0516	90.2334	94.4623	97.5199	98.9490	100.0000

C. Principal component analysis for adult male specimens of *Nyctibatrachus acanthodermis*, *N. gavi*, *N. grandis*, *N. indraneili*, *N. major*, *N. radcliffei* sp. nov. and *N. sylvaticus*.

Variable	PC 1	PC 2	PC 3	PC 4	PC 5	PC 6	PC 7	PC 8	PC 9
HW/HL	-0.274630	0.173544	**0.753449**	0.537276	-0.060599	-0.175778	-0.002154	0.027980	0.052460
SL/HL	**-0.701400**	-0.565509	-0.116102	0.281214	-0.005835	0.218936	-0.189159	-0.076284	0.078165
EL/HL	-0.444968	**-0.628317**	0.331681	-0.458070	0.167583	-0.203717	0.010943	-0.132366	0.012465
HL/SVL	0.407343	**0.850528**	-0.173341	-0.166931	0.008501	-0.094717	-0.127765	-0.087264	0.140638
FAL/SVL	-0.589046	0.557333	0.311254	-0.226048	-0.399836	0.071448	-0.119190	-0.055983	-0.110067
HAL/SVL	**-0.769195**	-0.117697	-0.363589	-0.076614	-0.461483	-0.089548	0.161665	0.037206	0.088956
TL/SVL	**-0.813944**	0.143684	-0.418792	0.041858	0.218335	-0.223755	-0.166055	0.108293	-0.051709
SHL/SVL	**-0.663811**	0.550458	-0.229279	0.288102	0.256256	0.032851	0.176048	-0.145726	-0.042473
FOL/SVL	**-0.695370**	0.366584	0.353361	-0.348302	0.276549	0.192543	0.058253	0.118098	0.070756
Eigenvalue	3.456633	2.250603	1.303120	0.868477	0.594515	0.230645	0.154532	0.083167	0.058309
% Total variance	38.40703	25.00670	14.47911	9.64974	6.60572	2.56272	1.71702	0.92408	0.64787
Cum Eigenvalue	3.456633	5.707236	7.010356	7.878833	8.473347	8.703993	8.858524	8.941691	9.000000
Cumulative %	38.4070	63.4137	77.8928	87.5426	94.1483	96.7110	98.4280	99.3521	100.0000
