# Supplementary material for: Seven new species of Night Frogs (Anura, Nyctibatrachidae) from the Western Ghats Biodiversity Hotspot of India, with remarkably high diversity of diminutive forms
Source: PeerJ. 2017 Feb 21;5:e3007. doi: 10.7717/peerj.3007 (PMC5322763; doi:10.7717/peerj.3007)
Supplement: Table S4 [file peerj-05-3007-s006.pdf]

Supplemental information: **Tables**

Seven new species of Night Frogs (Anura, Nyctibatrachidae) from the Western Ghats Biodiversity Hotspot of India, with remarkably high diversity of diminutive forms

Sonali Garg, Robin Suyesh, Sandeep Sukesan and S D Biju

Table S4. Scores for Discriminant function analysis of principal components resulting from nine size-corrected morphometric variables of adult male specimens. Values in bold indicate variables with the highest loadings for discriminant function roots of eigenvalue >1.0.

**A**	Discriminant function analysis for *Nyctibatrachus anamallaiensis*, *N. beddomii*, *N. manalari* sp. nov., *N. minimus*, *N. pulivijayani* sp. nov., *N. robinmoorei* sp. nov. and *N. sabarimalai* sp. nov.					
Variable	DFA root 1	DFA root 2	DFA root 3	DFA root 4	DFA root 5	DFA root 6
PC 1	**-0.352661**	0.239231	-0.081456	**-0.399780**	-0.155440	-0.701170
PC 2	0.114722	**0.260795**	0.039562	0.054166	0.155402	-0.549966
PC 3	0.142055	0.020900	0.138590	**-0.545846**	-0.231257	-0.361661
PC 4	0.042257	**0.248925**	-0.193497	0.051665	0.075011	0.621444
PC 5	0.052861	-0.098553	**-0.516860**	**-0.382095**	-0.219623	0.160404
PC 6	0.007922	-0.087967	-0.033603	-0.143546	**0.636912**	-0.299520
PC 7	-0.027936	0.035192	0.140299	-0.222518	-0.050003	0.401124
PC 8	-0.011745	0.011621	0.164377	-0.197301	0.227887	0.521203
PC 9	0.019185	-0.023245	0.118341	0.078415	-0.357877	-0.042495
Eigenvalue	21.63428	10.51349	4.81371	2.289589	1.214652	0.103094
Cumulative %	53.32737	79.24258	91.10811	96.751827	99.745879	100.000000

**B**	Discriminant function analysis for *Nyctibatrachus athirappillyensis* sp. nov., *N. deccanensis*, *N. kempholeyensis*, *N. minor* and *N. webilla* sp. nov.			
Variable	DFA root 1	DFA root 2	DFA root 3	DFA root 4
PC 1	**-0.357711**	**-0.433683**	**0.470935**	0.298535
PC 2	0.193941	**-0.765537**	**-0.357402**	-0.163035
PC 3	-0.123361	0.043192	-0.090157	0.200764
PC 4	0.110317	-0.142839	0.427594	0.012992
PC 5	0.155428	-0.015700	-0.006147	0.673828
PC 6	-0.018058	-0.074957	0.087713	-0.545524
PC 7	0.071579	-0.053981	0.087064	0.176711
PC 8	0.136272	0.097187	0.201054	-0.417397
PC 9	0.096340	0.068704	0.294064	0.156414
Eigenvalue	6.32855	1.959712	1.527055	0.055717
Cumulative %	64.11231	83.965475	99.435546	100.000000

**C**	Discriminant function analysis for *Nyctibatrachus acanthodermis*, *N. gavi*, *N. grandis*, *N. major*, *N. radcliffei* sp. nov. and *N. sylvaticus*. *Nyctibatrachus indraneili* was excluded from DFA due to availability of a single representative.				
Variable	DFA root 1	DFA root 2	DFA root 3	DFA root 4	DFA root 5
PC 1	**0.176161**	-0.200293	0.126533	-0.118893	0.050477
PC 2	**0.353200**	**0.315082**	0.103382	0.223684	-0.209783
PC 3	0.080081	0.068302	**-0.503501**	**-0.319431**	0.024912
PC 4	-0.054796	**0.228662**	0.130158	**-0.560779**	0.363869
PC 5	-0.011191	0.059640	-0.261307	**0.375977**	0.131899
PC 6	-0.039757	0.180237	-0.110768	0.197928	-0.512070
PC 7	-0.005875	0.245499	**0.354062**	0.112894	-0.174500
PC 8	-0.010054	-0.144680	0.030043	-0.170434	-0.827268
PC 9	-0.005561	0.045541	0.042566	-0.103560	0.218363
Eigenvalue	17.92688	4.962967	2.406914	1.141375	0.066327
Cumulative %	67.63721	86.362237	95.443403	99.749751	100.000000
