# Supplementary material for: Seven new species of Night Frogs (Anura, Nyctibatrachidae) from the Western Ghats Biodiversity Hotspot of India, with remarkably high diversity of diminutive forms
Source: PeerJ. 2017 Feb 21;5:e3007. doi: 10.7717/peerj.3007 (PMC5322763; doi:10.7717/peerj.3007)
Supplement: Table S5 [file peerj-05-3007-s007.pdf]

Supplemental information: **Tables**

Seven new species of Night Frogs (Anura, Nyctibatrachidae) from the Western Ghats Biodiversity Hotspot of India, with remarkably high diversity of diminutive forms

Sonali Garg, Robin Suyesh, Sandeep Sukesan and S D Biju

Table S5. Classification matrices from the discriminant function analyses.

A Observed classification for adult male specimens of *Nyctibatrachus anamallaiensis*, *N. beddomii*, *N. manalari* sp. nov., *N. minimus*, *N. pulivijayani* sp. nov., *N. robinmoorei* sp. nov. and *N. sabarimalai* sp. nov.

Species	Percent correct	*N. anamallaiensis*	*N. beddomii*	*N. manalari*	*N. minimus*	*N. pulivijayani*	*N. robinmoorei*	*N. sabarimalai*
N. anamallaiensis	100	5	0	0	0	0	0	0
N. beddomii	100	0	10	0	0	0	0	0
N. manalari	100	0	0	5	0	0	0	0
N. minimus	100	0	0	0	14	0	0	0
N. pulivijayani	100	0	0	0	0	5	0	0
N. robinmoorei	100	0	0	0	0	0	2	0
N. sabarimalai	100	0	0	0	0	0	0	5
Total	100	5	10	5	14	5	2	5

B Observed classification for adult male specimens of *Nyctibatrachus athirappillyensis* sp. nov., *N. deccanensis*, *N. kempholeyensis*, *N. minor* and *N. webilla* sp. nov.

Species	Percent correct	*N. athirappillyensis*	*N. deccanensis*	*N. kempholeyensis*	*N. minor*	*N. webilla*
N. athirappillyensis	100	5	0	0	0	0
N. deccanensis	100	0	15	0	0	0
N. kempholeyensis	90.9	0	0	10	1	0
N. minor	33.3	0	0	3	2	1
N. webilla	100	0	0	0	0	4
Total	87.8	5	15	13	3	5

C Observed classification for adult male specimens of *Nyctibatrachus acanthodermis*, *N. gavi*, *N. grandis*, *N. major*, *N. radcliffei* sp. nov. and *N. sylvaticus*. *Nyctibatrachus indraneili* was excluded from DFA due to availability of a single representative.

Species	Percent correct	*N. acanthodermis*	*N. gavi*	*N. grandis*	*N. major*	*N. radcliffei*	*N. sylvaticus*
N. acanthodermis	100	4	0	0	0	0	0
N. gavi	100	0	2	0	0	0	0
N. grandis	100	0	0	3	0	0	0
N. major	100	0	0	0	7	0	0
N. radcliffei	100	0	0	0	0	5	0
N. sylvaticus	100	0	0	0	0	0	4
Total	100	4	2	3	7	5	4
