# Supplementary material for: Seven new species of Night Frogs (Anura, Nyctibatrachidae) from the Western Ghats Biodiversity Hotspot of India, with remarkably high diversity of diminutive forms
Source: PeerJ. 2017 Feb 21;5:e3007. doi: 10.7717/peerj.3007 (PMC5322763; doi:10.7717/peerj.3007)
Supplement: Table S6 [file peerj-05-3007-s008.pdf]

Supplemental information: **Tables**

Seven new species of Night Frogs (Anura, Nyctibatrachidae) from the Western Ghats Biodiversity Hotspot of India, with remarkably high diversity of diminutive forms

Sonali Garg, Robin Suyesh, Sandeep Sukesan and S D Biju

Table S6. Morphometric measurements (in mm) of the adult specimens of seven new *Nyctibatrachus* species described in the study. The range, mean, and standard deviation are given for each parameter. Male (M) and female (F).

	N. athirappillyensis		***N. manalari***	***N. pulivijayani***	***N. radcliffei***	***N. robinmoorei***		***N. sabarimalai***		***N. webilla***
	M (*N*=5)	F (*N*=1)	M (*N*=5)	M (*N*=5)	M (*N*=5)	M (*N*=2)	F (*N*=1)	M (*N*=5)	F (*N*=1)	M (*N*=4)
SVL	20.9–22.8 (21.8±0.7)	24.1	13.1–15.4 (14.3±0.9)	13.3–14.9 (14.3±0.6)	32.8–38.3 (34.4±2.2)	12.2–13.4 (12.8±0.8)	17.4	12.3–13.2 (12.8±04)	17.1	18.7–20.7 (19.6±0.8)
HW	7.6–7.9 (7.8±0.1)	8.3	5.0–6.2 (5.7±0.5)	5.1–5.7 (5.4±0.3)	13.2–14.5 (13.7±0.5)	4.0–5.2 (4.6±0.8)	6.8	4.2–4.4 (4.3±0.1)	5.6	7.2–8.1 (7.6±0.5)
HL	7.7–8.2 (7.9±0.2)	8.2	4.2–5.1 (4.6±0.4)	5.0–5.8 (5.4±0.3)	12.0–13.6 (12.6±0.6)	4.1–5.2 (4.7±0.8)	5.5	4.9–5.3 (5.1±0.2)	4.4	6.4–6.9 (6.6±0.2)
SL	3.3–3.7 (3.5±0.1)	4.1	1.8–2.4 (2.1±0.2)	2.1–2.5 (2.3±0.2)	5.3–6.2 (5.7±0.4)	2.0–2.1 (2.1±0.1)	2.5	1.9–2.0 (2.0±0.1)	2.4	3.0–3.3 (3.1±0.2)
EL	2.3–2.7 (2.4±0.2)	2.7	1.4–1.8 (1.6±0.1)	1.5–1.8 (1.6±0.1)	3.6–3.9 (3.7±0.2)	1.5–1.5 (1.5±0)	1.8	1.4–1.6 (1.4±0.1)	1.9	2.2–2.5 (2.4±0.1)
EN	1.3–1.4 (1.4±0.1)	1.6	0.6–0.8 (0.7±0.1)	0.7–0.9 (0.8±0.1)	1.8–2.6 (2.1±0.3)	1.0–1.1 (1.1±0.1)	0.8	0.6–0.9 (0.8±0.1)	0.9	1.3–1.5 (1.4±0.1)
NS	1.4–1.7 (1.6±0.1)	1.9	0.9–1.1 (1.0±0.1)	1.0–1.3 (1.2±0.1)	2.6–3.3 (2.9±0.3)	1.2–1.3 (1.3±0.1)	1.3	0.9–1.2 (1.1±0.2)	1.1	1.5–1.9 (1.6±0.2)
IUE	2.5–2.9 (2.7±0.2)	3.1	1.9–2.3 (2.1±0.2)	1.9–2.3 (2.0±0.2)	3.4–3.9 (3.7±0.2)	1.9–2.0 (2.0±0.1)	2.1	1.6–2.0 (1.8±0.2)	2.2	2.6–3.0 (2.8±0.2)
UEW	1.1–1.4 (1.2±0.1)	1.3	0.7–1.0 (0.8±0.1)	0.7–0.9 (0.8±0.1)	1.7–2.3 (2.0±0.2)	0.5–0.6 (0.6±0.1)	0.7	0.6–0.8 (0.7±0.1)	0.9	0.9–1.2 (1.1±0.1)
IN	1.5-1.9 (1.7±0.2)	1.9	1.5–1.7 (1.6±0.1)	1.6–1.9 (1.7±0.1)	2.6-3.1 (2.9±0.2)	1.5–1.5 (1.5±0.0)	1.5	1.3–1.6(1.5±0.1)	1.6	2.0-2.2 (2.1±0.1)
FAL	3.6–3.8 (3.7±0.1)	4.4	2.0–2.5 (2.3±0.2)	2.3–2.8 (2.6±0.2)	6.5–6.9 (6.6±0.2)	1.9–2.0 (2.0±0.1)	2.7	2.4–2.8 (2.5±0.2)	3.2	3.3–3.6 (3.4±0.1)
HAL	5.3–5.8 (5.5±0.2)	6.2	3.3–3.9 (3.5±0.3)	3.0–3.6 (3.3±0.2)	9.5–11.2 (10.1±0.7)	1.7–1.9 (1.8±0.1)	3.6	2.5–2.9 (2.7±0.2)	3.3	4.6–4.9 (4.8±0.1)
TL	10.0–10.6 (10.2±0.2)	11.6	6.1–6.8 (6.5±0.3)	6.3–7.1 (6.7±0.4)	17.6–19.0 (18.3±0.5)	6.5–6.8 (6.7±0.2)	8.1	6.1–6.7 (6.3±0.2)	7.3	9.0–9.9 (9.3±0.4)
SHL	10.2–10.6 (10.3±0.2)	11.5	6.2–6.8 (6.5±0.2)	6.5–6.9 (6.7±0.1)	16.4–17.6 (16.9±0.5)	5.1–5.2 (5.2±0.1)	7.1	6.1–6.6 (6.3±0.2)	7.2	8.1–9.0 (8.5±0.4)
FOL	10.0–10.7 (10.4±0.3)	11.8	7.2–7.5 (7.4±0.2)	6.5–6.8 (6.6±0.1)	16.7–17.1 (16.9±0.2)	5.2–5.3(5.3±0.1)	7.2	6.1–6.6 (6.3±0.2)	6.9	9.0–9.8 (9.3±0.4)
