# Supplementary material for: Seven new species of Night Frogs (Anura, Nyctibatrachidae) from the Western Ghats Biodiversity Hotspot of India, with remarkably high diversity of diminutive forms
Source: PeerJ. 2017 Feb 21;5:e3007. doi: 10.7717/peerj.3007 (PMC5322763; doi:10.7717/peerj.3007)
Supplement: Table S7 [file peerj-05-3007-s009.pdf]

Supplemental information: **Tables**

Seven new species of Night Frogs (Anura, Nyctibatrachidae) from the Western Ghats Biodiversity Hotspot of India, with remarkably high diversity of diminutive forms

Sonali Garg, Robin Suyesh, Sandeep Sukesan and S D Biju

Table S7. Diagnostic characters for the new and morphologically related *Nyctibatrachus* species. (1) snout to vent size (SVL); (2) dorsal skin texture and dorsolateral folds; (3) inverted 'Y'-shaped ridge over the snout; (4) foot webbing; (5) third finger disc morphology; (6) fourth toe disc morphology; (7) relative size of finger disc compared to finger width; (8) relative size of toe disc compared to toe width; (9) HW/HL ratio; (10) TL/SHL ratio. All comparative measures are for adult male specimens.

	(1)	(2)	(3)	(4)	(5)	(6)	(7)	(8)	(9)	(10)
N. anamallaiensis	Miniature, 13.1–15.7 mm	Prominently shagreened with glandular projections, presence of weakly developed dorsolateral folds	Weakly developed	Absent	With dorso-terminal groove, cover bifurcate distally	With dorso-terminal groove, cover bifurcate distally	Slightly wide, FD_{III} 0.3, FW_{III} 0.2	Slightly wide, TD_{IV} 0.4, TW_{IV} 0.2–0.3	Head wider, 115.7–126.1%	Nearly equal, 98.4–101.6%
N. beddomii	Miniature, 13.3–18 mm	Smooth to shagreened with scattered spinular projections	Weakly developed	Absent	With dorso-terminal groove, cover bifurcate distally	With dorso-terminal groove, cover bifurcate distally	Slightly wide, FD_{III} 0.4–0.6, FW_{III} 0.2–0.3	Slightly wide, TD_{IV} 0.5–0.6, TW_{IV} 0.2–0.4	Head wider, 106.6–129.3%	Nearly equal, 95.9–101.6%
N. manalari	Miniature, 13.1–15.4 mm	Shagreened with scattered glandular projections, presence of weakly developed dorsolateral folds	Weakly developed	Absent	With dorso-terminal groove, cover bifurcate distally	With dorso-terminal groove, cover bifurcate distally	Slightly wide, FD_{III} 0.3–0.4, FW_{III} 0.2–0.3	Slightly wide, TD_{IV} 0.4–0.6, TW_{IV} 0.2–0.3	Head wider, 115.7–135.6%	Nearly equal, 98.4–101.5%
N. minimus	Miniature, 10–14 mm	Shagreened with faint and interrupted glandular projections	Weakly developed	Absent	With dorso-terminal groove, cover bifurcate distally	With dorso-terminal groove, cover bifurcate distally	Slightly wide, FD_{III} 0.3–0.4, FW_{III} 0.1–0.3	Slightly wide, TD_{IV} 0.4–0.5, TW_{IV} 0.2–0.3	Head nearly equal or wider, 95.7–108%	Thigh shorter or nearly equal, 82.4–98.5%
N. pulivijayani	Miniature, 13.3–14.9 mm	Shagreened with scattered glandular projections, presence of weakly developed dorsolateral folds	Indistinct	Absent	With dorso-terminal groove, cover bifurcate distally	With dorso-terminal groove, cover bifurcate distally	Slightly wide, FD_{III} 0.3–0.4, FW_{III} 0.2–0.3	Slightly wide, TD_{IV} 0.4–0.5, TW_{IV} 0.2–0.3	Nearly equal, 98.3–102%	Nearly equal, 94–104.5%
N. robinmoorei	Miniature, 12.2–13.4 mm	Shagreened with scattered glandular projections, presence of weakly developed dorsolateral folds	Weakly developed	Absent	With dorso-terminal groove, cover bifurcate distally	With dorso-terminal groove, cover bifurcate distally	Slightly wide, FD_{III} 0.4–0.5, FW_{III} 0.3–0.4	Slightly wide, TD_{IV} 0.5–0.6, TW_{IV} 0.3	Nearly equal, 97.6–100%	Thigh longer, 127.5–130.8%
N. sabarimalai	Miniature, 12.3–13.2 mm	Shagreened with scattered glandular projections, presence of weakly developed dorsolateral folds	Weakly developed	Absent	With dorso-terminal groove, cover bifurcate distally	With dorso-terminal groove, cover bifurcate distally	Slightly wide, FD_{III} 0.3–0.4, FW_{III} 0.2–0.3	Slightly wide, TD_{IV} 0.5–0.6, TW_{IV} 0.3–0.4	Head longer, 82.4–89.8%	Nearly equal, 98.4–101.6%
N. athirappillyensis	Small-sized,	Weakly wrinkled with	Well	Medium,	With dorso-	With dorso-	Slightly wide,	Moderately wide,	Nearly equal,	Nearly equal,

	20.9–22.8 mm	spinular projections	developed	$2^{+}IV2^{+}$	terminal groove, cover rounded distally	terminal groove, cover notched distally	FD_{III} 0.5–0.7, FW_{III} 0.3–0.4	TD_{IV} 0.7–0.8, TW_{IV} 0.3–0.5	96.3–100%	98–100%,
N. deccanensis	Miniature to small-sized, 16.1–20.8 mm	Weakly wrinkled with glandular ridges	Well developed	Medium, 3IV3	Without dorso-terminal groove	With dorso-terminal groove, cover bifurcate distally	Slightly wide, FD_{III} 0.4–0.5, FW_{III} 0.2–0.4	Slightly wide, TD_{IV} 0.4–0.9, TW_{IV} 0.2–0.6	Head wider, 101.3–125.8%	Thigh nearly equal or longer 92.9–115.8%
N. kempholeyensis	Miniature to small-sized, 15.5–21.6 mm	Weakly wrinkled with scattered granular projections and glandular ridges	Well developed	Medium, $2^{1}/_{2}IV2^{1}/_{2}$	With dorso-terminal groove, cover rounded distally	With dorso-terminal groove, cover rounded distally	Slightly wide, FD_{III} 0.4–0.7, FW_{III} 0.2–0.4	Moderately wide, TD_{IV} 0.7–1.0, TW_{IV} 0.3–0.5	Nearly equal, 98.9–106%	Thigh nearly equal or longer, 92.9–104.3%
N. minor	Miniature to small-sized, 15.4–17.9 mm	Weakly wrinkled with dorsolateral glandular ridges separated by an 'X' pattern on the anterior half	Well developed	Basal, 4^{-} $IV4^{-}$	With dorso-terminal groove, cover bifurcate distally	With dorso-terminal groove, cover bifurcate distally	Slightly wide, FD_{III} 0.4–0.5, FW_{III} 0.2–0.3	Slightly wide, TD_{IV} 0.4–0.6, TW_{IV} 0.2–0.3	Nearly equal or wider, 95.8–116.9%	Thigh nearly equal or longer 94.8–108.2%
N. webilla	Small-sized, 18.7–20.7 mm	Shagreened to granular with scattered glandular ridges	Well developed	Basal, $4^{-}IV4^{-}$	Without dorso-terminal groove	With dorso-terminal groove, cover bifurcate distally	Slightly wide, FD_{III} 0.3–0.4, FW_{III} 0.2–0.3	Moderately wide, TD_{IV} 0.6–0.7, TW_{IV} 0.3–0.4	Head wider, 110.8–117.4%	Thigh longer, 106.9–111.1%
N. acanthodermis	Large-sized, 52.9–66.2 mm	Prominently wrinkled with sharp spinular projections	Well developed	Large, $1^{1}/_{2}IV1^{1}/_{2}$	Without dorso-terminal groove	With dorso-terminal groove, cover rounded distally	Prominently wide, FD_{III} 1.4–1.6, FW_{III} 0.5–0.8	Prominently wide, TD_{IV} 1.9–2.6 , TW_{IV} 0.8	Head wider, 109.7–112.6%	Nearly equal, 99.3–100.3%,
N. gavi	Large-sized, 49.5–57.5 mm	Wrinkled with scattered spinular projections	Well developed	Large, 2IV2	Without dorso-terminal groove	With dorso-terminal groove, cover rounded distally	Prominently wide, FD_{III} 1.2–1.8, FW_{III} 0.6–0.8	Prominently wide, TD_{IV} 1.2–1.4, TW_{IV} 0.4–0.5	Head wider, 105.1–107.8%	Thigh longer, 102.6–112.1%
N. grandis	Large-sized, 62.2–76.9 mm	Wrinkled with scattered glandular projections	Well developed	Large, $1^{3}/_{4}IV1^{3}/_{4}$	Without dorso-terminal groove	With dorso-terminal groove, cover rounded distally	Prominently wide, FD_{III} 1.6–1.9, FW_{III} 0.5–0.8	Prominently wide, TD_{IV} 2.2–2.8, TW_{IV} 0.5–0.6	Head wider, 106.8–108.3%	Thigh longer, 104.3–113.2%
N. indraneili	Large-sized, 42.5 mm	Weakly wrinkled without glandular projections	Well developed	Large, $2^{-}IV2^{-}$	Without dorso-terminal groove	Without dorso-terminal groove	Moderately wide, FD_{III} 1.1, FW_{III} 0.7	Prominently wide, TD_{IV} 1.2, TW_{IV} 0.6	Head wider, 104.2%	Thigh longer, 110.5%
N. major	Medium-sized, 31.5–48.8 mm	Wrinkled with a few glandular warts	Well developed	Large, 2IV2	Without dorso-terminal groove	With dorso-terminal groove, cover rounded distally	Prominently wide, FD_{III} 0.8–1.0, FW_{III} 0.4–0.7	Prominently wide, TD_{IV} 0.9–1.4, TW_{IV} 0.4–0.7	Head wider, 110.5–117.8%	Nearly equal, 99.5–101.3%,
N. radcliffei	Medium-sized, 32.8–38.3 mm	Weakly wrinkled without prominent spinular projections	Well developed	Large, $1^{+}IV1$	Without dorso-terminal groove	With dorso-terminal groove and cover rounded distally	Prominently wide, FD_{III} 1.0–1.2, FW_{III} 0.4–0.6	Prominently wide, TD_{IV} 1.5–1.6, TW_{IV} 0.5–0.6	Head wider, 106.6–113.3%	Thigh longer, 107.1–110.2%
N. sylvaticus	Medium-sized, 28.6–36.2 mm	Wrinkled with scattered granular projections	Well developed	Large, 2IV2	With dorso-terminal groove, cover rounded distally	With dorso-terminal groove, cover rounded distally	Moderately wide, FD_{III} 0.6–0.9, FW_{III} 0.4–0.5	Prominently wide, TD_{IV} 0.9–1.1, TW_{IV} 0.4–0.5	Head wider, 109.3–116.7%	Thigh longer, 107.8–115.3%
