# Supplementary material for: Seven new species of Night Frogs (Anura, Nyctibatrachidae) from the Western Ghats Biodiversity Hotspot of India, with remarkably high diversity of diminutive forms
Source: PeerJ. 2017 Feb 21;5:e3007. doi: 10.7717/peerj.3007 (PMC5322763; doi:10.7717/peerj.3007)
Supplement: Table S8 [file peerj-05-3007-s010.pdf]

Supplemental information: **Tables**

Seven new species of Night Frogs (Anura, Nyctibatrachidae) from the Western Ghats Biodiversity Hotspot of India, with remarkably high diversity of diminutive forms

Sonali Garg, Robin Suyesh, Sandeep Sukesan and S D Biju

Table S8. Call properties of seven *Nyctibatrachus* species measured from single calls.

	N. athirappillyensis		*N. beddomii*		*N. manalari*	*N. minimus*	*N. minor*	*N. sabarimalai*	*N. webilla*
Call Parameters	Part 1	Part 2	Part 1	Part 2					
Duration (ms)	93.5	595.8	22.7	80.6	51.1	139.9	261.1	37.4	201.5
Rise time (ms)	43.6	1.7	3.6	20.6	6.7	1.9	40.5	1.3	52.9
Fall time (ms)	7.3	593.5	18.2	58.5	34.7	137.9	217.7	35.9	92.5
Pulses per call	-	25	-	9.0	-	6.0	18.0	-	20.0
Pulse rate (pulses/s)	-	42.2	-	110.0	-	45.6	70.8	-	104.0
Dominant frequency (kHz)	1.6	3.4	2.2	2.2	3.6	4.4	4.9	4.0	3.4
