# Supplementary material for: Seven new species of Night Frogs (Anura, Nyctibatrachidae) from the Western Ghats Biodiversity Hotspot of India, with remarkably high diversity of diminutive forms
Source: PeerJ. 2017 Feb 21;5:e3007. doi: 10.7717/peerj.3007 (PMC5322763; doi:10.7717/peerj.3007)
Supplement: Data S1 [file peerj-05-3007-s011.pdf]

Supplemental information: **Data**

Seven new species of Night Frogs (Anura, Nyctibatrachidae) from the Western Ghats Biodiversity Hotspot of India, with remarkably high diversity of diminutive forms

Sonali Garg, Robin Suyesh, Sandeep Sukesan and S D Biju

Data S1. **Call descriptions for three previously known *Nyctibatrachus* species.**

***Nyctibatrachus beddomii*.** The male of *N. beddomii* was observed to produce a single type of call with two distinct parts, hereafter termed part 1 and part 2. The calls were not delivered in groups and the two call parts had a fixed order, i.e. part 1 followed by part 2. Part 1 of the call was comprised of a single pulse while part 2 had a pulsatile temporal structure. The entire call had a duration of 131.2 ms and an overall dominant frequency of 2.2 KHz. Part 1 had a call duration of 22.7 ms, fall time of 18.2 ms and was without a significant rise time (3.6 ms). Part 2 of the call was longer in duration (80.6 ms) compared to part 1 and was comprised of 9 pulses delivered at a rate of 110.0 pulses/s. The amplitude envelope of part 2 was characterized by a rise time of 20.6 ms, fall time of 58.5 ms and also showed two distinct dominant frequency peaks (Table S8; Figs. S2A–S2D).

***Nyctibatrachus minimus*.** The male of *N. minimus* was observed to produce a single type of call with pulsatile temporal structure. Calls were not delivered in groups and had uniform intervals. A single call had a duration of 261.1 ms and was comprised of 18 pulses delivered at a rate of 70.8 pulses/s. Amplitude envelope of the call was characterized by a rise time of 40.5 ms, fall time of 217.7 ms and the overall dominant frequency of 4.9 KHz with a single broad peak (Table S8; Figs. S2E–S2H).

***Nyctibatrachus minor*.** The male of *N. minor* was observed to produce a single type of call. The calls had a single pulse and were not delivered in groups. A typical advertisement call had a duration of 37.4 ms. Amplitude envelope of the call was characterized by a rise time of 1.3 ms, fall time of 35.9 ms and the overall dominant frequency of 4.0 KHz with three distinct distinct frequency peaks (Table S8; Figs. S2I–S2L).
